# Assessing the Clinical Efficacy of a Virtual Reality Tool for the Treatment of Obesity: Randomized Controlled Trial

**DOI:** 10.2196/51558

**Published:** 2024-04-05

**Authors:** Dimitra Anastasiadou, Pol Herrero, Paula Garcia-Royo, Julia Vázquez-De Sebastián, Mel Slater, Bernhard Spanlang, Elena Álvarez de la Campa, Andreea Ciudin, Marta Comas, Josep Antoni Ramos-Quiroga, Pilar Lusilla-Palacios

**Affiliations:** 1 Department of Clinical and Health Psychology, Universitat Autònoma de Barcelona Bellaterra, Barcelona Spain; 2 Psychiatry, Mental Health and Addictions Research Group, Vall d´Hebron Research Institute Barcelona Spain; 3 RE-FiT Barcelona Research Group, Vall d’Hebron Research Institute & Parc Sanitari Pere Virgili Barcelona Spain; 4 Virtual Bodyworks S.L. Barcelona Spain; 5 The Institute of Neurosciences, Universitat de Barcelona Barcelona Spain; 6 Department of Clinical Psychology and Psychobiology, Universitat de Barcelona Barcelona Spain; 7 Endocrinology and Nutrition Department, Vall d’Hebron University Hospital Barcelona Spain; 8 Vall d’Hebron Research Institute, Universitat Autònoma de Barcelona Barcelona Spain; 9 Centro de Investigación Biomédica en Red de Diabetes y Enfermedades Metabólicas Asociadas, Instituto de Salud Carlos III Madrid Spain; 10 Psychiatry Department, Vall d’Hebron University Hospital Barcelona Spain; 11 Department of Psychiatry and Legal Medicine, Universitat Autònoma de Barcelona Barcelona Spain; 12 Biomedical Network Research Centre on Mental Health (CIBERSAM) Barcelona Spain

**Keywords:** obesity, virtual reality, psychological treatment, embodiment, motivational interviewing, self-conversation

## Abstract

**Background:**

Virtual reality (VR) interventions, based on cognitive behavioral therapy principles, have been proven effective as complementary tools in managing obesity and have been associated with promoting healthy behaviors and addressing body image concerns. However, they have not fully addressed certain underlying causes of obesity, such as a lack of motivation to change, low self-efficacy, and the impact of weight stigma interiorization, which often impede treatment adherence and long-term lifestyle habit changes. To tackle these concerns, this study introduces the VR self-counseling paradigm, which incorporates embodiment and body-swapping techniques, along with motivational strategies, to help people living with obesity effectively address some of the root causes of their condition.

**Objective:**

This study aims to assess the clinical efficacy of ConVRself (Virtual Reality self-talk), a VR platform that allows participants to engage in motivational self-conversations.

**Methods:**

A randomized controlled trial was conducted with 68 participants from the bariatric surgery waiting list from the obesity unit of the Vall d’Hebron University Hospital in Barcelona, Spain. Participants were assigned to 1 of 3 groups: a control group (CG), which only received treatment as usual from the obesity unit; experimental group 1 (EG1), which, after intensive motivational interviewing training, engaged in 4 sessions of VR-based self-conversations with ConVRself, and underwent embodiment and body-swapping techniques; and experimental group 2 (EG2), which engaged in 4 VR-based sessions led by a virtual counselor with a prerecorded discourse, and only underwent the embodiment technique. In the case of both EG1 and EG2, the VR interventions were assisted by a clinical researcher. Readiness to change habits, eating habits, and psychological variables, as well as adherence and satisfaction with ConVRself were measured at baseline, after the intervention, 1 week after the intervention, and 4 weeks after the intervention.

**Results:**

Regarding the primary outcomes, EG1 (24/68, 35%) and EG2 (22/68, 32%) showed significant improvements in *confidence to lose weight* compared to the CG (22/68, 32%) at all assessment points (β=−.16; *P*=.02). Similarly, EG1 demonstrated a significant increase after the intervention in *readiness to exercise more* compared to the CG (β=−.17; *P*=.03). Regarding the secondary outcomes, EG1 participants showed a significant reduction in *uncontrolled eating* (β=.71; *P*=.01) and *emotional eating* (β=.29; *P*=.03) compared to the CG participants, as well as in their *anxiety* levels compared to EG2 and CG participants (β=.65; *P*=.01). In addition, participants from the experimental groups reported high adherence and satisfaction with the VR platform (EG1: mean 59.82, SD 4.00; EG2: mean 58.43, SD 5.22; *d*=0.30, 95% CI −0.30 to 0.89).

**Conclusions:**

This study revealed that using VR self-conversations, based on motivational interviewing principles, may have benefits in helping people with obesity to enhance their readiness to change habits and self-efficacy, as well as reduce dysfunctional eating behaviors and anxiety.

**Trial Registration:**

ClinicalTrials.gov NCT05094557; https://www.clinicaltrials.gov/study/NCT05094557

## Introduction

### Background

Obesity is defined as a severe, chronic, complex, and multifactorial disease with detrimental effects on the individual’s physical and psychological health [1]. Various treatment options are currently available for obesity, including psychological interventions, behavioral interventions for lifestyle modification, pharmacotherapy, and bariatric surgery (BS) [2,3]. All these treatments are taken into consideration in a process of shared decision-making to generate a patient-centered plan [4].

According to the National Institute for Health and Care Excellence [5], BS is a treatment option available for people living with obesity with BMI >40 kg/m^2^ (or BMI between 35 kg/m^2^ and 40 kg/m^2^ if relevant comorbidity is present that could be improved with weight loss). People living with obesity who undergo BS require rigorous and comprehensive preoperative and postoperative monitoring and support. This support should emphasize the adoption and maintenance of a healthy lifestyle, as well as the identification of any clinical or psychological barriers that may hinder adherence to postoperative treatment [5]. However, when assessing the long-term maintenance of weight loss in obesity management, including through BS procedures, achieving lasting and sustainable changes in body composition remains highly challenging [6]. Therefore, for more effective obesity management, it is beneficial to incorporate multicomponent psychological interventions that aim to improve health as a whole, foster self-efficacy and self-esteem, and prioritize sustainable goals chosen by the individual [2].

In this context, motivational interviewing (MI) has gained considerable recognition as an effective approach to enhance treatment adherence [7] and has been included in the most recent psychological and behavioral recommendations for obesity management [2]. Specifically, MI is a counseling technique based on a person-centered approach that aims to help individuals to identify the discrepancies between their goals and current circumstances and empower them to explore new alternatives toward behavior change, thereby enhancing motivation and resolving ambivalence [8].

Furthermore, in recent years, telemental health care has emerged as a rapid and efficient means of establishing different communication channels between patients and mental health professionals and has been proven capable of transforming the availability, accessibility, and efficacy of psychological treatments [9,10], particularly during the COVID-19 pandemic and beyond [11]. Recent research findings provide support for the acceptability, feasibility, and preliminary effectiveness of using new technologies in the treatment of eating and weight disorders [12-14]. Specifically, the integration of virtual reality (VR) into psychological interventions for these conditions holds great promise in addressing some of the factors associated with the development or maintenance of the disorders. Through the provision of multisensory experiences, extrinsic feedback, and opportunities for embodiment, psychological VR interventions, which are rooted primarily in cognitive behavioral therapy principles, have demonstrated effectiveness as complementary tools in managing obesity and have been associated with promoting behavior changes and weight reduction, reducing binge eating episodes, and addressing body image concerns [12,14-18]. While these interventions focus on correcting specific behaviors and provide patients with a safe context to practice eating, emotional, and relational management, they do not adequately address some of the root causes of obesity, such as a lack of motivation to change, low self-efficacy, and the impact of weight stigma interiorization, which are factors that often hinder treatment adherence and long-term maintenance of lifestyle habits [19,20].

The SOCRATES project (Self Conversation in Virtual Reality Embodiment to Enhance Healthier Lifestyles Among People with Obesity), funded by the European Commission (951930), has introduced the VR self-counseling paradigm, referred to as ConVRself (Virtual Reality self-talk), which uses embodiment and body-swapping techniques. On the one hand, embodiment enables participants to experience the perceptual illusion that the virtual body is their own [21,22]. On the other hand, body swapping allows embodying alternatingly between 2 virtual bodies (or *avatars*, eg, one representing the self and the other a counselor) and maintaining a conversation between these 2 embodied perspectives [23-25]. In this study, ConVRself is used as a solution to help people living with obesity explore some of the root causes of their condition. Drawing on MI principles, this approach allows participants to engage in self-counseling through motivational conversations with themselves. In particular, participants are immersed in a VR environment that resembles a counselor’s office. First, they embody an avatar that looks like themselves (a *look-alike*), and from this perspective, they explain their problem, goals, and/or aspirations to a counselor, seated in front of them across a table. Once they have stated their problem, participants switch to embody the counselor’s avatar. In this new perspective, they listen to a playback of their own words and watch the related body gestures made during their speech. After having listened, they can respond from the counselor’s embodied perspective. In this way, participants engage in a self-conversation by adopting 2 different embodied perspectives and maintaining a conversation between them. To ensure that these conversations remain motivational, participants undergo intensive training in MI before engaging in the VR self-counseling experience. The objective of these virtual self-conversations has been to address the following challenges that people living with obesity face: (1) to raise awareness of their actual condition, (2) to better understand and address the impact of weight bias interiorization, and (3) to increase their self-efficacy by setting realistic goals in line with their values.

### Objectives

In this randomized controlled trial (RCT) based on the protocol described in the study by Anastasiadou et al [26], our primary objective was to evaluate the clinical efficacy of the ConVRself platform under 3 different conditions: one that uses the embodiment and body-swapping techniques together with the MI training, one only using the embodiment technique, and a group receiving treatment as usual (TAU).

We hypothesized that participants who used ConVRself with embodiment and body-swapping techniques, along with the MI training, would show greater improvement in the primary outcomes (motivation to lose weight and exercise more) as well as the secondary outcomes (lifestyle habits and psychological well-being) from baseline (T0) to the 3 postintervention assessments compared to participants who used ConVRself with only the embodiment element or the TAU group.

## Methods

### Ethical Considerations

This study was approved by the Clinical Research Ethics Committee and Research Projects Committee of the Vall d’Hebron University Hospital (VHUH). The study protocol was preregistered at ClinicalTrials.gov (NCT05094557) and published at *BMJ Open* [26]. Before enrollment in the study, written informed consent was obtained from all participants. To maintain confidentiality, each participant was assigned a numerical code. No compensation was provided for participating in the study.

### Recruitment

**Figure 1:**
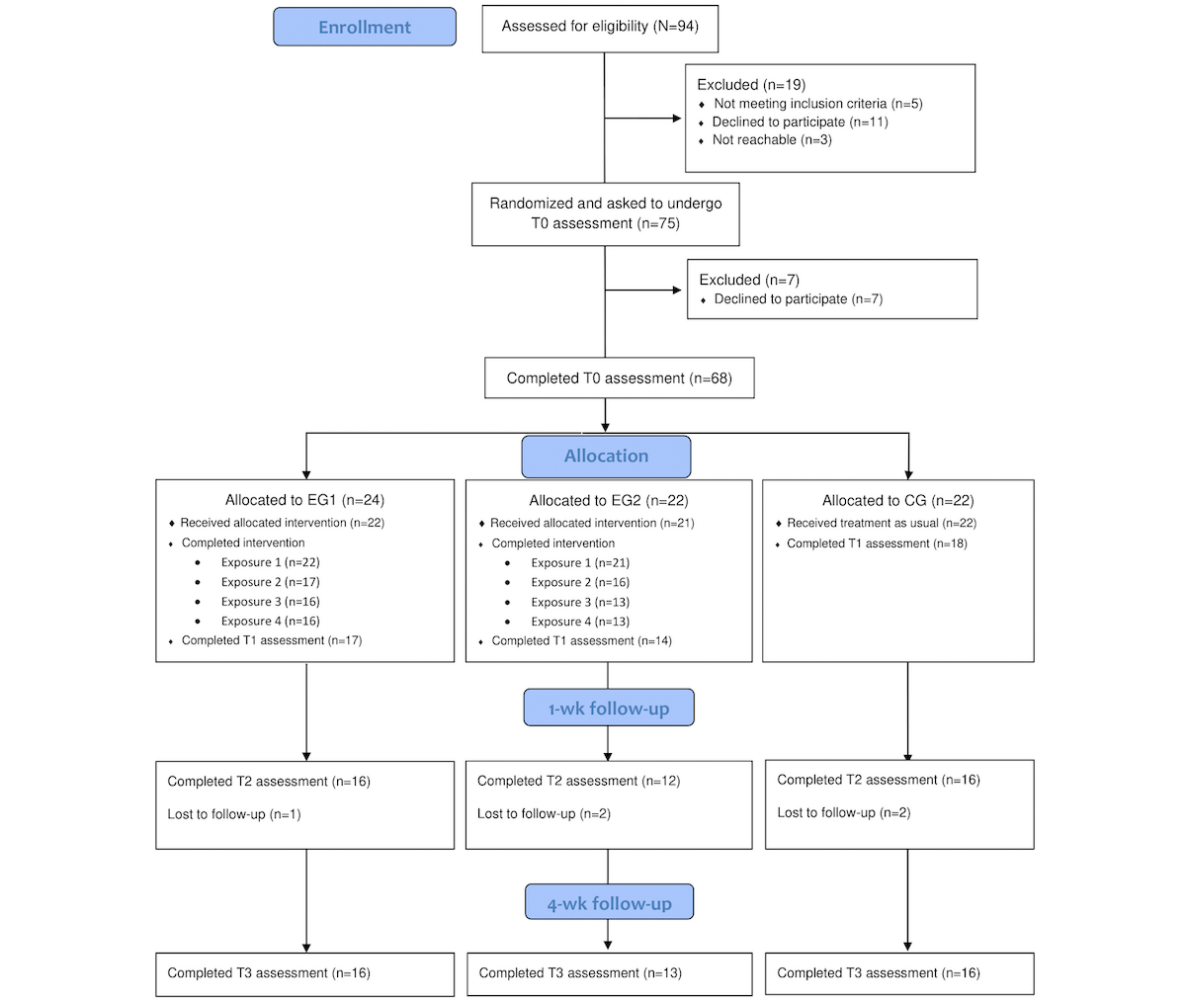
Flow of participants through each stage of the randomized controlled trial. CG: control group; EG1: experimental group 1; EG2: experimental group 2; T0: baseline; T1: after the intervention; T2: 1 week after the intervention; T3: 4 weeks after the intervention.

Participants from the BS waiting list from the obesity unit of the VHUH within the national health system were assessed for eligibility between December 2021 and April 2023. To be eligible for inclusion, participants had to meet the following criteria: aged between 18 and 65 years; BMI ≥30 kg/m^2^; receiving ambulatory treatment at the VHUH; not undergoing any other concurrent treatment specifically related to their obesity condition from other centers; possessing minimal digital skills, which means being able to use a digital device (smartphone, tablet, or computer) to make telephone calls and have video conversations via the internet, send or receive emails, and search for information about products and (health) services; demonstrable oral and written understanding of the Spanish language; and willingness to provide informed consent to participate. As stated in the usability study conducted by Anastasiadou et al [27], the BMI criterion was revised to include only a minimum threshold of ≥30 kg/m^2^ for eligibility to participate in the study, contrary to the BMI criteria originally set in the study protocol (BMI between ≥30 kg/m^2^ and ≤55 kg/m^2^) [26]. Participants were not eligible if they met ≥1 of the following exclusion criteria: presence of an eating disorder in the last 2 years, nonstabilized severe mental disorder that could interfere with the successful implementation of the research protocol (ie, psychosis, depression with suicidal risk, alcohol or drug abuse, and psychotic or manic symptoms), intellectual disability or any major illness seriously affecting cognitive performance (ie, neurological disorders), and personal history of epilepsy (to avoid the potential risk of triggering seizures in this population). Of the 94 participants assessed for eligibility, 68 (72%) were recruited to participate in the study (Figure 1). Details regarding the sample size calculation are available in the protocol (32).

### Design

We conducted an RCT with 3 parallel groups (experimental group 1 [EG1], experimental group 2 [EG2], and a control group [CG]) with a 1:1:1 allocation ratio. Measurements were carried out at 4 time points: T0, after the intervention (T1), 1 week after the intervention (T2), and 4 weeks after the intervention (T3).

### Procedure

Participant data were managed and automatically distributed using REDCap (Research Electronic Data Capture; Vanderbilt University) [28,29], hosted at the Vall d’Hebron Research Institute in Barcelona, Spain. The data collected at T0, T1, T2, and T3 were obtained through web-based self-report instruments completed by participants using REDCap. In addition, the research team members measured participants’ weight and height to calculate their BMI.

Potential eligible participants for the project were referred to the research team by health care professionals from the VHUH obesity unit. These participants received TAU provided by the obesity unit, which included regular medical, nutritional, and psychiatric follow-ups conducted by specialists at the hospital. Eligible participants were contacted via telephone by a research team member who provided information about the study. If they agreed to participate in the study, an appointment was scheduled at the hospital. During this appointment, a clinical interview was conducted by the team member to confirm the participants’ eligibility. Sociodemographic and clinical variables were also collected, and weight and height measurements were taken. Once the necessary data were collected, participants were randomly distributed in 1 of the 3 groups (EG1, EG2, and CG) using REDCap and were then informed about the outcome of the randomization. Next, they were asked to complete the web-based T0 assessment within the following week, which was managed by the automatic email distribution facilitated by REDCap. In addition, participants were asked to watch a 30-minute video with psychoeducational advice created by the research team. This video provided information about obesity and the promotion of healthy lifestyle habits. It was shared to ensure that all participants had a similar background concerning the concept of obesity and some knowledge about healthy lifestyle habits.

After the completion of the T0 assessment, participants received their assigned interventions based on their respective groups for a period of 10 weeks. The timeline of the assessments is depicted in Multimedia Appendix 1. The T1 assessment took place during week 6, T2 assessment during week 7, and T3 assessment during week 10 using the same automatic email distribution as with the T0 assessment. Furthermore, REDCap implemented an automatic alert system to remind participants to complete the questionnaires if they had not done so at the scheduled time. Finally, participants who underwent BS during the course of the 4 exposures (for details, refer to the Experimental Groups subsection) were still requested to respond to the T1 assessment, even if they did not complete the full intervention.

### Interventions

#### CG Participants

After completing the T0 assessment, CG participants did not receive any intervention besides the TAU provided by the VHUH obesity unit and the psychoeducational video.

#### Experimental Groups

##### Interventions Overview

After completing the T0 assessment, EG1 participants underwent a 1-day MI training at the hospital facilities. The training lasted 4 hours and focused on developing basic MI skills. In addition, participants had an individual coaching session via telephone 1 week after the training. The initial in-person training session was led by an expert in MI (author PLP), while the follow-up sessions were carried out by the other members of the research team. At the end of the initial session, 3 photographs (2 from the front and 1 from the side) were taken of each participant to create their look-alike avatars, while their counselor’s avatar was designed according to each participant’s preferences concerning sex, age, and body shape. EG2 participants did not receive any MI training. Instead, they were invited to the hospital facilities where a research team member took photographs for the creation of their avatars.

Two weeks later, EG1 and EG2 participants engaged in 4 VR scenarios at the hospital facilities, assisted by a team clinical researcher. Scenarios were distributed in weekly sessions, each lasting 30 minutes, over a period of 4 weeks. After each exposure, satisfaction and adherence to the VR experiments were assessed for both groups using a semistructured interview designed by the research team. In addition, several self-report questionnaires were administered to the participants, including the readiness ruler (RR), the Suitability Evaluation Questionnaire (SEQ), and the Body Ownership Questionnaire (BOQ). For more detailed information, refer to the Measures subsection.

Specifically, the exposures of the 2 groups are detailed in the following subsections.

##### EG1 Participants

###### Overview

In each of the 4 scenarios, participants had a self-conversation using the embodiment and body-swapping techniques. Specifically, for exposures 1, 2, and 4, participants alternated between their look-alike avatar and the avatar of a counselor. During exposure 3, they alternated between their own avatar and an avatar representing their future self—a representation depicting their future self after adopting a healthier lifestyle 5 years from the present. When embodying the counselor and the future-self avatars, participants applied the MI techniques they had learned during the intensive training.

###### Exposure 1: Embodied Discussion About Problems and Solutions

The purpose of this scenario was to facilitate motivational self-conversations between the participant and their counselor about the lifestyle changes they planned to achieve in terms of eating healthier and being more physically active.

###### Exposure 2: Overcoming Self-Stigmatization

The objective of this intervention was to explore and address the participants’ subjective weight stigma experiences and their interiorization through motivational self-conversations between the participant and their counselor.

###### Exposure 3: Illustrating the Possibility of Autonomy

The objective of this intervention was to explore, through motivational self-conversations with participants’ future selves, how these future selves successfully achieve the goals that participants set in the present and to identify any barriers encountered during the process.

###### Exposure 4: Summing Up

Participants started their self-conversations by sharing the insights they gained from the previous exposures. In addition, they reflected on how these insights could be effectively implemented in their daily lives.

##### EG2 Participants

###### Overview

EG2 participants received a traditional counseling approach in a virtual setting. In all 4 exposures, participants were only embodied in their own look-alike avatars. First, participants engaged in a prerecorded discourse, conducted by a virtual counselor, that posed open-ended questions to which the participants responded. Second, the virtual counselor provided general and prerecorded advice that could be beneficial for the participants in promoting a healthier lifestyle.

###### Exposure 1: Embodied Discussion About Problems and Solutions

For this exposure, the virtual counselor asked about the perceived barriers that participants faced when trying to adopt a healthier lifestyle and then provided practical recommendations to help overcome these barriers and facilitate the adoption of a healthier lifestyle. Examples of these recommendations are as follows:

Thank you for sharing your goals with me. Next, I’m going to give you some simple tips that can help improve your lifestyle and overall well-being. First of all, try to avoid miracle diets as none of them work in the medium and long term...Second, make healthy choices regarding your diet. Certain foods should be prioritized, others limited, and some replaced with healthier alternatives.

###### Exposure 2: Overcoming Self-Stigmatization

In this exposure, the participants shared their subjective experiences of body size discrimination, and the virtual counselor offered practical advice about how to deal with them. Some examples of the advice given are as follows:

Despite the enticing advertisements encouraging you to believe that image is everything, never forget that your appearance is just one aspect of who you are. Try to develop your sense of identity based on all the things you can do and the person you are deep within, despite inhabiting a larger body...Secondly, appreciate and take care of your body—a body that, when healthy, can accomplish many things.

###### Exposure 3: Illustrating the Possibility of Autonomy

In this exposure, the conversation focused on discussing the potential positive effects that people living with obesity may experience when they adopt healthier behaviors that prioritize their overall health, rather than solely focusing on weight. An example of such a conversation is as follows:

Given that many factors influence your health status, some of which are beyond your control, one important step you can take to promote good health while living in a larger body is to adopt healthy eating and exercise habits, along with activities that foster social support, without solely focusing on weight loss.

###### Exposure 4: Summing Up

Participants engaged in a conversation during which the virtual counselor provided a general summary of the main concepts explained in the previous exposures.

### Technical Features of the VR System

The VR system used in the study consisted of both hardware and software components. The VR hardware used was the Meta Quest 2, which is a stand-alone headset developed by Reality Labs (Meta Platforms, Inc). The main part of the hardware consists of a head-mounted display worn by the participants. This head-mounted display has a vision- and inertia-based inside-out tracking system that allows precise tracking of the user’s head movements. In addition, the Meta Quest 2 comes equipped with hand controllers that enable interaction within the virtual environment. Finally, the Meta Quest 2 also incorporates a built-in processor that generates stereoscopic images and spatialized audio. The headset runs the ConVRself application, which was developed using the Unity 3D development environment (Unity Technologies).

### ConVRself Software

The VR software ConVRself, developed by Virtual Bodyworks SL, displays 3D scenarios and virtual human representations. This VR application enables participants to have self-conversations by embodying their look-alike avatar and another avatar alternatingly. The software generates scenarios that have 3 stages: calibration, tutorial, and experience. In the first stage, calibration, participants wore a VR headset and held VR controllers. The system used this setup to calculate an internal human representation that synchronized the movements of the embodied avatar with the participants’ own movements. During the next 2 stages, tutorial and experience, participants were immersed in the virtual environment, embodying their look-alike avatar from a first-person perspective. To enhance the sense of embodiment, they could see themselves reflected in a virtual mirror on their left. To watch a video showing how ConVRself works, please refer to the supplementary materials in the study by Anastasiadou et al [26]. Specifically in the tutorial stage, participants had to follow detailed audio instructions provided by the application to get used to the platform before the actual experience. In addition, during this stage, participants underwent the embodiment technique to foster the illusion that the virtual body represented their own. Finally, in the experience stage, participants engaged in different exposures.

To minimize the risks associated with COVID-19, the research team followed a safety protocol that included wearing masks, carrying out regular hand disinfection, and using the CX1 decontamination system (Cleanbox Technology) to clean the Meta Quest 2 headsets and controllers used in the study.

### Measures

#### Primary Outcomes

The primary outcomes (motivation to lose weight and exercise more) were assessed using 2 measurement tools: the RR [8] and the Stages of Change Questionnaire for Weight Management (S-Weight) and Processes of Change Questionnaire for Weight Management (P-Weight) [30]. The RR is a visual analog scale ranging from 1 to 10 that assessed participants’ readiness for, confidence about, and perception of the importance of changing behavior with regard to 2 specific areas: (1) achieving a healthy weight and (2) exercising more. The S-Weight questionnaire consists of 5 mutually exclusive items that aim to allocate participants to 1 of the 5 stages of change in weight management according to the transtheoretical model: precontemplation, contemplation, preparation, action, and maintenance. The P-Weight questionnaire is a 5-point Likert scale (ranging from *strongly disagree* to *strongly agree*) consisting of 34 items developed to assess 4 processes of change for weight management: (1) emotional re-evaluation (13 items), (2) weight management actions (7 items), (3) environmental restructuring (5 items), and (4) weight consequences evaluation (9 items). The scores of each subscale were summed to obtain a total score and were then transformed on a new scale ranging from 0 to 100. The Spanish version of P-Weight showed an adequate internal consistency (Cronbach α coefficients ranged from 0.78 to 0.96 in both individuals with normal weight and individuals with overweight and obesity) [30]. The Cronbach α values in this study for the P-Weight subscales were 0.74 for emotional re-evaluation, 0.74 for weight management actions, 0.76 for environmental restructuring, and 0.76 for weight consequences evaluation.

#### Secondary Outcomes

##### Eating Habits

The Three-Factor Eating Questionnaire–Revised 18 items (TFEQ-R18) [31] is a self-report questionnaire designed to measure 3 aspects of eating behavior: (1) cognitive restraint (CR; 6 items), (2) uncontrolled eating (UE; 9 items), and (3) emotional eating (EE; 3 items). Participants responded to each item on a 4-point Likert scale ranging from *definitely true* to *definitely false*. The total scores of each subscale were obtained by summing the scores of individual items. The Spanish version of the TFEQ-R18 [32] showed good internal consistency (Cronbach α coefficients ranged from 0.75 to 0.87) in a sample of young and healthy adults. The Cronbach α values in this study for each subscale were 0.59 for CR, 0.87 for UE, and 0.78 for EE.

The Eating Habits Questionnaire [33] is a self-report questionnaire with 37 items, each rated using a 5-point Likert scale ranging from *never* to *always*. This questionnaire measures eating habits across 8 different spheres: (1) sugar intake (4 items), (2) healthy eating (9 items), (3) physical activity (3 items), (4) diet caloric intake (5 items), (5) psychological well-being (3 items), (6) types of aliments (5 items), (7) knowledge and control (5 items), and (8) alcohol intake (2 items). A total score was obtained as the average of the scores from the 8 spheres. The Cronbach α coefficient for the complete questionnaire was 0.87 and ranged from 0.58 to 0.94 for the different spheres in a Spanish sample of adult participants living with overweight and obesity [33]. In this study, the Cronbach α value was 0.88.

##### Psychological Variables

Psychological functioning was estimated with the Hospital Anxiety and Depression Scale (HADS) [34]. The HADS is a self-report 14-item questionnaire (7 items for anxiety and 7 items for depression). Participants rated each item on a 4-point Likert scale to indicate the presence and severity of anxiety and depression symptoms. The total scores of each factor were obtained by summing the scores of individual items. The Spanish version showed high internal consistency, with a Cronbach α value of 0.86 for the 2 factors in a sample of patients and healthy controls [35]. The Cronbach α values in this study were 0.77 for the depression subscale and 0.79 for the anxiety subscale.

Body satisfaction was measured using the 10-item validated Spanish version of the Body Shape Questionnaire [36]. This self-report scale is rated using a 6-point Likert scale ranging from *never* to *always*. A total score was obtained by summing the scores of individual items. In this study, the Cronbach α value for this questionnaire was 0.89.

The Modified Weight Bias Internalization Scale (WBIS-M) [37] was used to assess weight bias interiorization. The WBIS-M is a self-report 11-item unidimensional scale rated using a 7-point scale ranging from *strongly disagree* to *strongly agree*. A total score was obtained as the sum of the scores of individual items. The Cronbach α coefficient for the complete questionnaire ranged from 0.93 to 0.94 in a sample of Spanish adults [37]. The Cronbach α value for this questionnaire in this study was 0.86.

The Cognitive Reserve Questionnaire [38] was used to measure participants’ cognitive reserve. This self-report questionnaire consists of 8 items that evaluate aspects generally related to cognitive reserve, such as educational status (own and parental), occupational status, completion of training courses, musical training, and language proficiency. The total score was obtained by summing the item scores. The Cronbach α value for this questionnaire in this study was 0.63.

##### Adherence and Satisfaction Regarding VR Experiments

To measure satisfaction, acceptance, and security regarding the use of the ConVRself platform, we used the SEQ [39]. The SEQ is a 14-item questionnaire, with 13 items rated on a 5-point Likert scale ranging from *not at all* to *very much*, as well as a last open-ended question where participants can provide suggestions and additional feedback. For the specific purposes of this study, the word “rehabilitation” in item 11 was replaced by “obesity treatment.” The total score was obtained by summing the scores of the first 13 items. Validation studies of the SEQ showed an acceptable internal consistency, with a Cronbach α value of 0.70 in samples of individuals with different physical pathologies. The Cronbach α value of the SEQ in this study was 0.61.

The BOQ evaluates the subjective illusion of body ownership in a VR context through a 7-point Likert scale ranging from *strongly disagree* to *strongly agree*. Specifically, the 4 questions of this scale were obtained from a previous study evaluating ConVRself [25]. The questionnaire assesses body ownership when (1) looking down at the virtual body, (2) observing oneself in a virtual mirror, (3) perceiving body movements, and (4) recognizing oneself. These questions were asked for the participants’ own avatar as well as the counselor’s avatar (except for the question regarding the self-recognition item, which was only asked for the participants’ own avatar). For EG2 participants, the questions were asked exclusively for their own avatar because they did not experience the body-swapping technique. For more information about particular items, please refer to the protocol published in the study by Anastasiadou et al [26].

Along with the questionnaires, a brief interview was conducted after each exposure to assess participants’ satisfaction with the VR experience and acceptability of ConVRself.

### Statistical Analysis

Initial analyses involved comparisons of EG1, EG2, and CG participants on sociodemographic and clinical characteristics, adherence (dropout analysis), and assessment variables at the T0 level. Subsequent assessments examined those participants who completed the long-term follow-up assessment and those who did not on the same sociodemographic and clinical characteristics and outcome variables at T0. Depending on variable types or objective, various statistical tests were used: the Shapiro-Wilk test for assessing the normality of the distribution, a 1-way ANOVA with group as a factor for normally distributed variables, the Friedman test for variables with non-normal distributions, and the chi-square test for qualitative variables.

To handle missing data within questionnaires, passive multiple imputation was used. This approach updates total scores based on recent imputed values at the item level, thereby ensuring complete data for analysis [40,41].

Analyses for our primary and secondary outcomes were tested with 2-level hierarchical linear models (HLMs). These models were implemented with group (EG1, EG2, and CG) and time (T0, T1, T2, and T3 for the RR; T0, T1, and T3 for other variables) as fixed factors and participants nested within time as a random factor. For model adjustment, we used restricted maximum likelihood as the estimation method and the scaled identity as the error covariance structure. Potential moderators (such as age, sex, BMI at T0, time of treatment at the VHUH obesity unit, the presence of physical comorbidities, current mental illness, and Cognitive Reserve Questionnaire scores at T0) were examined. All covariables were grand mean centered.

An intent-to-treat (ITT) analysis was conducted using the available data of all participants for outcome estimation. This analysis, leveraging the ability of HLMs to integrate missing data, offers a more realistic, unbiased analysis compared to traditional methods [42]. To ensure the robustness of the results obtained from the primary and secondary ITT analyses, we also conducted a per-protocol analysis. This analysis included only participants who completed the RCT. Results from the per-protocol analysis were reported if they differed from the results of the aforementioned ITT analyses.

Effect sizes were calculated and reported using *R*^2^ marginal (variance explained by the fixed effects) and *R*^2^ conditional (variance explained by the entire model, both fixed and random effects) [43,44] for HLMs and Cohen *d* for comparison between the experimental groups in VR technique. Their magnitude was interpreted according to the Cohen guidelines where *R*^2^=0.01 or *d*≤0.2 represents a small effect, *R*^2^=0.06 or *d*=0.5 represents a medium effect, and *R*^2^≥0.14 or *d*≥0.8 represents a large effect [45].

Analyses were conducted using SPSS (version 29.0; IBM Corp) and RStudio (version 2022.12.0; Posit Software, PBC), with the *mice* package from RStudio [46] for passive multiple imputation. A 2-tailed significance level of .05 was applied to all statistical tests.

## Results

### Sample Description

As shown in Figure 1, of the initial 94 participants assessed for the study, 19 (20%) were excluded before the randomization, resulting in 75 (80%) participants being enrolled and randomized. However, after receiving the T0 assessment, of the 75 participants, 7 (9%) decided not to continue participating; therefore, 68 (91%) participants completed the T0 assessment and were allocated to 1 of the 3 groups: EG1 (n=24, 35%), EG2 (n=22, 32%), and CG (n=22, 32%).

In EG1, of the 24 participants, 22 (92%) received the allocated intervention, of whom 16 (73%) completed the 4 exposures. At T1, 77% (17/22) responded; and at T2 and T3, 73% (16/22) responded. In EG2, of the 22 participants, 21 (95%) received the allocated intervention, of whom 13 (62%) completed the 4 exposures. At T1, 67% (14/21) responded; at T2, 57% (12/21); and at T3, 62% (13/21). Regarding the CG, of the 22 participants, at T1, 18 (82%) responded; at T2, 16 (73%); and at T3, 16 (73%). The adherence analysis did not reveal statistical differences among the groups (*χ*^2^_6_*=*4.6; *P*=.60).

The sociodemographic and clinical characteristics of the sample are presented in Multimedia Appendix 2. Participants had a mean age of 44.22 (SD 10.30) years, a mean BMI of 43.58 (SD 5.96) kg/m^2^, and had been receiving treatment at the VHUH obesity unit for an average of 21.62 (SD 11.18) months. Most of the participants were female individuals (54/68, 79%), Spanish citizens (51/68, 75%), employed either part time or full time (39/68, 57%), and lived with their family (50/68, 74%). Regarding clinical data, most of the participants had physical comorbidities (58/68, 85%), with pain and cardiovascular problems being the most prevalent, and no current mental illness (53/68, 78%). When comparing all groups on sociodemographic variables, significant differences were found regarding sex (*χ*^2^_2_=8.4; *P*=.02), the presence of current mental illness (*χ*^2^_2_=8.4; *P*=.02), and physical comorbidities (*χ*^2^_2_=6.0; *P*=.05).

Furthermore, there were no differences among the groups in any assessment variable at the T0 level (*P*>.05). Descriptive results of the primary and secondary outcomes of all participants, and separately for each group, are presented in Multimedia Appendices 3 and 4.

When comparing participants who completed the long-term follow-up assessments and those who did not, significant results were found regarding the following variables: (1) age (t_65_=−2.68; *P*=.009), (2) TFEQ-R18 CR (t_66_=−2.74; *P*=.008), (3) Eating Habits Questionnaire total score (t_64_=−3.23; *P*=.002), and (4) S-Weight (*χ*^2^_3_=16.3; *P*=.001). More precisely, participants with a higher mean age (46.4, SD 10.3) were more likely to complete the follow-up assessments than younger participants (mean age 39.7, SD 8.8). In addition, participants who completed all assessment points had significantly higher means in the TFEQ-R18 CR subscale and Eating Habits Questionnaire total score, and a higher percentage of them were at the maintenance stage (S-Weight), compared to those who did not complete all assessments.

### Primary Outcomes

#### Overview

The results of the analysis examining the effects of group condition and time are shown in Table 1. The HLMs revealed significant results for some RR scales, while no effects were observed for group versus time and time for P-Weight and S-Weight.

**Table 1:** Results from the hierarchical linear models^a^ for each variable^b^.

Variable		β (SE)	*t* test (*df*)	*P* value	*R*^2^ marginal^c^		*R*^2^ conditional^d^	
**RR^e^: importance of losing weight**						0.099		0.583
	Time	.17 (.11)	1.61 (182)	.11				
	Time×group	−.05 (.05)	−1.00 (186)	.34				
**RR** **: importance of exercising more**						0.122		0.523
	Time	.33 (.17)	1.95 (188)	.05				
	Time×group	−.07 (.08)	−0.96 (190)	.34				
**RR** **: confidence to lose weight**						0.151		0.727
	Time	.52 (.15)	3.47 (169)	<.001				
	Time×group	−.16 (.07)	−2.34 (172)	.02				
**RR** **: confidence to exercise more**						0.124		0.623
	Time	.55 (.18)	3.01 (179)	.003				
	Time×group	−.16 (.08)	−1.89 (183)	.06				
**RR** **: readiness to lose weight**						0.126		0.778
	Time	.36 (.15)	2.48 (160)	.01				
	Time×group	−.10 (.07)	−1.56 (163)	.12				
**RR** **: readiness to exercise more**						0.119		0.649
	Time	.60 (.17)	3.54 (177)	.001				
	Time×group	−.17 (.08)	−2.21 (181)	.03				
**P-Weight^f^: emotional re-evaluation**						0.165		0.712
	Time	−1.05 (1.03)	0.45 (120)	.31				
	Time×group	.21 (.47)	−0.36 (124)	.66				
**P-Weight: weight management actions**						0.154		0.667
	Time	−.06 (.71)	−0.09 (124)	.93				
	Time×group	.21 (.32)	0.66 (128)	.51				
**P-Weight:** **environmental restructuring**						0.136		0.750
	Time	1.07 (.62)	1.72 (116)	.09				
	Time×group	−.46 (.28)	−1.64 (119)	.10				
**P-Weight:** **weight consequences evaluation**						0.212		0.717
	Time	.38 (0.86)	0.44 (122)	.66				
	Time×group	−.16 (.39)	−0.42 (125)	.67				
**S-Weight^g^**						0.084		0.559
	Time	−.22 (.11)	−1.92 (121)	.06				
	Time×group	.10 (.05)	1.92 (125)	.06				
**TFEQ-R18^h^** **:** **cognitive restraint**						0.106		0.610
	Time	.39 (.47)	0.82 (126)	.41				
	Time×group	.08 (.21)	0.39 (130)	.70				
**TFEQ-R18:** **uncontrolled eating**						0.068		0.801
	Time	−1.82 (.62)	−2.93 (107)	.004				
	Time×group	.71 (.28)	2.53 (109)	.01				
**TFEQ-R18: emotional eating**						0.070		0.772
	Time	−.83 (.30)	−2.80 (110)	.006				
	Time×group	.29 (.13)	2.15 (112)	.03				
**Eating Habits Questionnaire total score**						0.104		0.838
	Time	.09 (.05)	1.88 (101)	.06				
	Time×group	−.003 (.02)	−0.14 (103)	.90				
**HADS^i^** **: anxiety**						0.209		0.735
	Time	−1.24 (.57)	−2.18 (117)	.03				
	Time×group	.65 (.30)	2.55 (120)	.01				
**HADS** **: depression**						0.239		0.735
	Time	−1.07 (.51)	−2.09 (118)	.04				
	Time×group	.44 (.23)	1.92 (121)	.06				
**BSQ-10^j^**						0.170		0.844
	Time	−.91 (1.29)	−0.70 (105)	.50				
	Time×group	.46 (.58)	0.79 (107)	.43				
**WBIS-M^k^**						0.165		0.805
	Time	−.004 (.15)	−0.03 (110)	.98				
	Time×group	−.02 (.07)	−0.34 (112)	.73				

^a^All hierarchical linear models were estimated with time and group as fixed effects; participant nested time as random effects; and age, sex, BMI at baseline, time of treatment at the obesity unit of the hospital, the presence of physical comorbidities, current mental illness, and Cognitive Reserve Questionnaire scores at baseline as covariables.

^b^Numbers presented are estimated values.

^c^*R*^2^ marginal refers to the amount of variance explained by the fixed effects.

^d^*R*^2^ conditional refers to the amount of variance explained by the entire model, both fixed and random effects.

^e^RR: readiness ruler.

^f^P-Weight: Processes of Change Questionnaire for Weight Management.

^g^S-Weight: Stages of Change Questionnaire for Weight Management.

^h^TFEQ-R18: Three-Factor Eating Questionnaire–Revised 18 items.

^i^HADS: Hospital Anxiety and Depression Scale.

^j^BSQ-10: Body Shape Questionnaire, 10-item version.

^k^WBIS-M: Modified Weight Bias Internalization Scale.

#### RR Analysis

**Figure 2:**
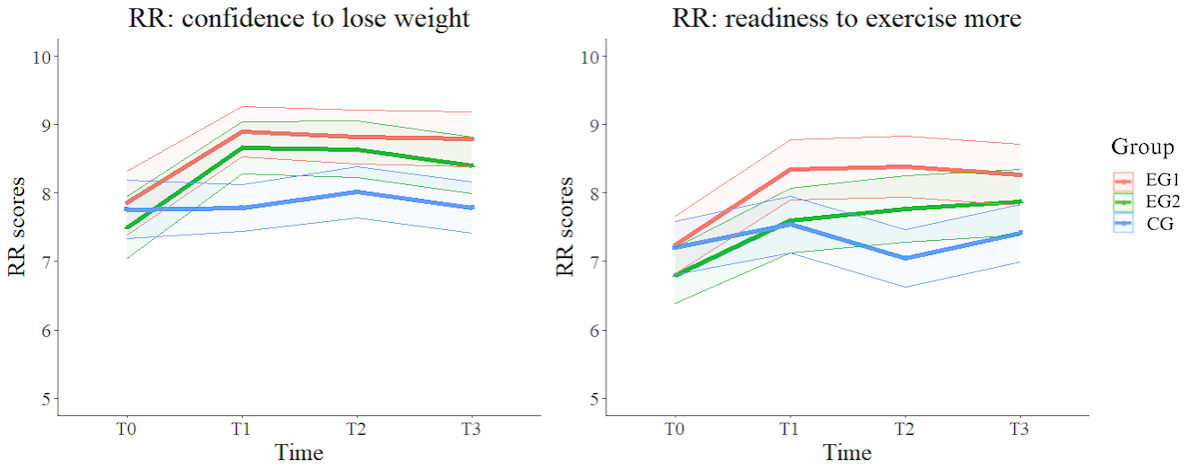
Estimated means of primary outcomes *confidence to lose weight* and *readiness to exercise more* in group versus time effect. The values are based on estimated means derived from hierarchical linear models with intent-to-treat analysis. Within each group, the central line represents the estimated mean outcome, while the surrounding error areas, delimited by 2 outer lines, illustrate the upper and lower bounds of the 95% CI around the estimated mean. CG: control group; EG1: experimental group 1; EG2: experimental group 2; RR: readiness ruler; T0: baseline; T1: after the intervention; T2: 1 week after the intervention; T3: 4 weeks after the intervention.

First, regarding *confidence to lose weight*, the HLM revealed a significant group versus time effect (β=−.16; *P*=.02). Post hoc comparisons revealed that both EG1 and EG2 showed significant differences compared to the CG at T0 versus T1, T0 versus T2, and T0 versus T3. This notable increase in *confidence to lose weight* for both groups can be seen in Figure 2. Second, a significant group versus time effect for *readiness to exercise more* (β=−.17; *P*=.03) was found, with post hoc comparisons showing a significant increase for EG1 compared to CG at T0 versus T2. The significant interaction effect is represented graphically in Figure 2, where we can also see how the different group conditions evolve through the different time measures. Finally, participants from all groups had a significant improvement in their *confidence to exercise more* between T0 and T1 and between T0 and T3 (β=.55; *P*=.003) and in their *readiness to lose weight* between T0 and T1, T0 and T2, and T0 and T3 (β=.36; *P*=.01).

### Secondary Outcomes

#### Overview

The HLMs revealed significant results for some TFEQ-R18 and HADS subscales, while no significant group versus time and time effects were found for the Eating Habits Questionnaire, Body Shape Questionnaire, and WBIS-M. The significant interaction effect for the TFEQ-R18 and HADS subscales is represented graphically in Multimedia Appendix 5.

#### TFEQ-R18 Analysis

The HLMs revealed a significant time×group effect for the UE (β=.71; *P*=.01) and EE subscales (β=.29; *P*=.03). Post hoc comparisons revealed consistently lower levels of UE for EG1 versus CG across all time measures, and for EE, a reduction for EG1 versus CG at T0 versus T3.

#### HADS Analysis

A significant group versus time effect was found for the anxiety subscale (β=.65; *P*=.01). In particular, post hoc comparisons revealed greater reductions in anxiety levels between T0 and T1 for EG1 compared to EG2 and CG. For the depression subscale, only a significant time effect was found (β=1.07; *P*=.04), indicating a decrease in depression levels across all groups over time.

The HLM per-protocol analysis revealed consistent results with the ITT analysis, showing no significant differences between the 2 approaches.

#### Adherence and Satisfaction Regarding VR Experiments (SEQ and BOQ)

For the SEQ, the experimental groups demonstrated high suitability scores with the VR platform (EG1: mean 59.82, SD 4.00; EG2: mean 58.43, SD 5.22; *d*=0.30, 95% CI −0.30 to 0.89).

Regarding the BOQ, both groups displayed average positive agreement scores, showing higher mean in EG1 than in EG2. Specifically, from the perspective of their look-alike avatar, for item 1 (looking down at the virtual body), EG1 had a mean of 2.09 (SD 1.17), while EG2 had a mean of 0.05 (SD 2.12; *d*=1.18, 95% CI 0.53-1.81); for item 2 (observing oneself in a virtual mirror), EG1 scored a mean of 2.34 (SD 0.70), and EG2 scored a mean of 1.53 (SD 1.58; *d*=0.66, 95% CI 0.05-1.26); for item 3 (perceiving body movements), EG1 scored a mean of 2.56 (SD 0.45), and EG2 scored a mean of 2.14 (SD 0.97; *d*=0.56, 95% CI −0.54 to 1.14); and for item 4 (recognizing oneself), EG1 scored a mean of 1.53 (SD 1.31), and EG2 scored a mean of 1.36 (SD 1.57; *d*=0.12, 95% CI −0.47 to 0.71). From the counselor’s perspective, EG1 also demonstrated average agreement scores: (1) looking down at the virtual body: mean 1.72 (SD 1.25); (2) observing oneself in a virtual mirror: mean 1.99 (SD 0.81); and (3) perceiving body movements: mean 2.25 (SD 0.64).

## Discussion

### Principal Findings

This study is focused on assessing the clinical efficacy of the ConVRself platform in tackling some of the root causes of obesity. The findings of this study confirmed our hypothesis, indicating that ConVRself with embodiment and body-swapping elements, along with MI training, significantly enhanced participants’ confidence to lose weight and readiness to perform physical exercise. In addition, this intervention proved effective in reducing dysfunctional eating behaviors and anxiety compared to the other groups. Overall, these findings are consistent with previous studies on the beneficial effects of positive, instructional, and motivational self-talk for performance [47].

The adherence analyses revealed that participants who had higher adherence to the treatment were those who reported, at T0, higher CR in relation to their eating habits, had healthier eating habits, and were in the *maintenance* stage of their change process, indicating a sustained commitment to lifestyle changes [8]. In line with these findings, a previous study showed that patients with obesity before BS who were more ready to limit food intake and were actively engaged in physical activity were more likely to adhere to dietary and physical activity recommendations after BS [48]. The dropout ratio of the study was 33.8% and was similar among the groups. This finding is aligned with another RCT that used VR psychological treatments for weight management [49], in which no differences between treatment conditions were found in the dropout rates.

As regards the sociodemographic information at T0, the average age closely aligns with findings from various studies involving patients who were on the BS waiting list or who had recently undergone surgery [50], as well as studies involving VR in the psychological management of obesity [51]. In addition, the uneven sex distribution in the study sample—79% (54/68) of the participants were female individuals—is common in studies with patients who have undergone BS [50,52,53], and also in VR interventions for weight management [49,51]. In fact, a recent systematic review that included 24 studies that used VR to treat obesity [15] reported that in 8 trials with both men and women, 93% of the sample were women. Several studies have explored this phenomenon [54,55], showing that women tend to experience higher body image dissatisfaction, psychological disturbances, and a greater desire to lose weight than men. This may explain why they are more frequently represented in clinical research studies.

Regarding clinical variables, consistent with previous literature [56], a high proportion of our sample (58/68, 85%) exhibited physical comorbidities, with pain, endocrine disorders, breathing problems, and cardiovascular problems being the most prevalent. These findings, together with the high prevalence of participants in our sample who were unemployed or on sick leave and with severe obesity (BMI: mean 43.58, SD 5.96 kg/m^2^), further confirm the debilitating nature of the disease. In addition, the most prevalent mental disorders observed were anxiety (8/68, 12%) and depression (6/68, 9%). However, neither disorder reached significant levels of morbidity based on the recommended cutoff points of the HADS [57]. Interestingly, our results indicate lower levels of anxiety and depression compared to previous research on patients who have undergone BS [58,59].

Regarding the assessment of motivation to change, measured along 3 dimensions (importance, confidence, and readiness) over time, participants initially reported a high importance placed on losing weight or exercising more. This posed challenges in detecting significant changes over time, as they were predominantly situated in the action and maintenance phases, as indicated by the S-Weight scores. However, all 3 groups improved over time in confidence to exercise more and readiness to lose weight. This was likely influenced by their positive expectations related to the BS or their mere involvement in the study. The participants’ expectation of undergoing BS, in alignment with recent literature [53], potentially served as an additional motivator in their personal journey toward change [60,61]. Notably, most participants had already consulted with the nutritionist and endocrinologist of the obesity unit on multiple occasions and had received instructions on how to implement modifications to their diet and exercise routines in preparation for surgery. The adherence to these recommendations provided valuable insights into their likelihood of being eligible to undergo surgery soon. Consequently, the findings regarding changes in motivation to change over time remain inconclusive, making it difficult to draw specific conclusions regarding postintervention improvements in these aspects.

Regarding the group versus time effect on motivation to change, the results indicated that participants who used ConVRself reported a significant increase in their confidence to lose weight and a higher readiness to engage in exercise compared to the CG. These positive effects were maintained for a duration of between 1 and 4 weeks after the exposures. This finding is particularly encouraging for behavior change, given that, for a person to initiate and maintain a change process, they must believe that the change is important and have confidence in their ability to achieve it [8]. Notably, increased confidence is often associated with a greater propensity to adopt self-regulation skills, including control over eating behaviors and improvement in physical activity [62]. In this sense, our results derived from the TFEQ-R18 suggested improved control over eating behaviors over time among the ConVRself group, as evidenced by lower tendencies in EE and UE behaviors, compared to the CG. Previous studies employing VR interventions with people living with obesity have yielded similar results, consistently demonstrating increased self-efficacy and readiness to initiate behavior change [63-65].

Apart from the aforementioned improvement in eating control in EG1, the same group also experienced a notable reduction in anxiety from T0 to T1 compared to EG2 and CG. These findings align with the results of 2 RCTs conducted by Manzoni et al [64,65]. In these studies, it was demonstrated that a relaxation treatment augmented by VR was more effective in reducing anxiety and EE behaviors than traditional meditation interventions at 2-week and 3-month follow-ups among women living with obesity. It is worth mentioning that anxiety scores in EG1 decreased more after the intervention compared to EG2 and CG; however, this reduction was not sustained over time. We believe that this result can be attributed to the absence of ongoing psychological support after the intervention period with ConVRself. This finding emphasizes how patients can be highly sensitive to changes in the treatment process during the preoperative period and underscores the constant need for psychological support in all pre- and postoperative treatment phases [5]. In this context, similar to the successful application of VR in addressing various mental disorders, including anxiety [66], ConVRself has the potential to provide an additional benefit by enabling patients to cope with anxiety and develop strategies aligned with their values.

In line with our previous usability study [27], high SEQ scores on ConVRself indicated high usability and acceptance of the platform by people living with obesity, which means that the platform was well adapted to this population. In addition, as regards the body ownership of the avatars, the results obtained are similar to those reported in previous literature [25,27], which indicates that participants experienced a strong sense of body ownership over the virtual avatars. However, inconsistent outcomes were found with EG2 for the *looking down at the virtual body* item compared to the other body ownership evaluations. The likely reason for this anomaly is a technological problem. Whenever participants looked down while embodied in their look-alike avatars, they could only see their knees. This limited visibility of their legs was primarily due to their anatomy (body size and shape), which caused most of their legs to be out of view. EG2 participants, particularly, failed to infer that seeing their knees implied seeing their entire legs. Despite the avatar’s anatomy being the same for all participants, we believe that EG1 participants were more focused on engaging in self-conversation, while EG2 participants placed greater emphasis on the physical appearance of their avatars.

### Limitations and Strengths

This study has several limitations. First, the high presence of physical comorbidities in the participants may have been influenced by the impact of delayed medical care and the exacerbation of conditions due to the COVID-19 pandemic [56]. This could potentially affect the generalizability of our results to the broader population. Second, a high dropout rate was observed, which made it challenging to achieve the expected adherence rate as stated in the study protocol [26]. This high dropout rate and the resulting smaller sample size may have impeded the detection of medium or small effects within the sample, particularly the potential differences among the groups. Third, the study design did not allow for a clear separation of the effects of ConVRself and the motivational training on the primary and secondary variables. The interpretations derived from our results could be attributed to either the effects of the motivational training or the virtual self-conversations, or, more likely, a combination of both. Fourth, the short follow-up period may limit the generalizability of the results. It becomes necessary to conduct a longer-term follow-up of the patients to observe whether the changes obtained with the ConVRself platform are sustained. Unfortunately, this was not feasible in this study due to time constraints imposed by the European SOCRATES project and the specific characteristics of our sample (patients on the BS waiting list). Furthermore, despite intensive basic training in MI, real competence in it demands constant and prolonged practice, potentially influencing the results. Finally, the high expectation of improvement from BS may have influenced the positive outcomes; therefore, it would be necessary to corroborate the results in a population with morbid obesity but without BS expectations.

The strengths of this study are its experimental design, specifically a study with 3 experimental groups and 4 assessment points, and the well-balanced distribution of the sample across groups. Moreover, we conducted a comprehensive evaluation of the participants’ health, including a clinical interview that considered both physical comorbidities and mental illness at T0. Furthermore, EG1 participants received intensive training before the VR intervention, which was led by an MI expert. In terms of statistical analysis, HLMs were used in conjunction with ITT analyses, enabling the inclusion of all available data from the study, including information from participants who dropped out. Finally, there are no previous studies on MI training aimed at patients rather than therapists, either in obesity or other medical fields. This opens up possibilities to train patients as experts, making them self-aware about their own condition and capable of self-motivation.

### Conclusions

In conclusion, using VR self-conversations to address the root causes of obesity has demonstrated important benefits and can be safely applied, with no side effects, among this population. In particular, the VR self-conversation with novel techniques of embodiment and body-swapping was well received by EG1 participants and was effective in enhancing self-efficacy and readiness to change, as well as in reducing dysfunctional eating behaviors and anxiety, compared to the other groups. Despite the apparent complexity of the procedures (self-conversation with embodiment and body-swapping), participants were able to complete the exposures, and they engaged in meaningful self-conversations about their obesity-related challenges and potential solutions. In this regard, a future study will provide qualitative data (currently under analysis and subject to another publication) on the unfolding of the motivational self-conversation process.

As for future perspectives, our findings underscore the importance of incorporating innovative psychological interventions to promote overall well-being and facilitate improvements in eating behaviors and lifestyle beyond mere weight loss. Such integrated interventions are crucial not only during the preoperative phase but also for the long-term maintenance of positive outcomes after BS. Future research should be conducted with ConVRself as a treatment not only for people living with obesity but also for patients with mental disorders or addictive behaviors. The potential of enriching virtual self-conversation during moments of blockage in patients with artificial intelligence techniques presents an exciting future research line.

## Appendix

Multimedia Appendix 1 Procedure and timeline of the randomized controlled trial.

Multimedia Appendix 2 Sociodemographic and clinical characteristics of all participants, and separately for each group.

Multimedia Appendix 3 Descriptive statistics (mean and SD) on scales and subscales of the study divided into groups and time measures.

Multimedia Appendix 4 Stages of Change Questionnaires for Weight Management frequencies and percentages divided into time and groups.

Multimedia Appendix 5 Estimated means of secondary outcomes in group versus time effect (intent-to-treat analysis).

Multimedia Appendix 6 CONSORT checklist.

## Abbreviations

BOQ: Body Ownership Questionnaire
BS: bariatric surgery
CG: control group
ConVRself: Virtual Reality self-talk
CR: cognitive restraint
EE: emotional eating
EG1: experimental group 1
EG2: experimental group 2
HADS: Hospital Anxiety and Depression Scale
HLM: hierarchical linear model
ITT: intent-to-treat
MI: motivational interviewing
P-Weight: Processes of Change Questionnaire for Weight Management
RCT: randomized controlled trial
REDCap: Research Electronic Data Capture
RR: readiness ruler
SEQ: Suitability Evaluation Questionnaire
S-Weight: Stages of Change Questionnaire for Weight Management
T0: baseline
T1: after the intervention
T2: 1 week after the intervention
T3: 4 weeks after the intervention
TAU: treatment as usual
TFEQ-R18: Three-Factor Eating Questionnaire–Revised 18 items
UE: uncontrolled eating
VHUH: Vall d’Hebron University Hospital
VR: virtual reality
WBIS-M: Modified Weight Bias Internalization Scale

## Data Availability

The data sets generated and analyzed during this study are available from the corresponding author on reasonable request.

## References

[1] (2021). Obesity and overweight. World Health Organization.

[2] Vallis M, Macklin D, Russell-Mayhew S (2020). Effective psychological and behavioural interventions in obesity management. Adult Obesity Clinical Practice Guidelines.

[3] Wharton S, Lau DC, Vallis M, Sharma AM, Biertho L, Campbell-Scherer D, Adamo K, Alberga A, Bell R, Boulé N, Boyling E, Brown J, Calam B, Clarke C, Crowshoe L, Divalentino D, Forhan M, Freedhoff Y, Gagner M, Glazer S, Grand C, Green M, Hahn M, Hawa R, Henderson R, Hong D, Hung P, Janssen I, Jacklin K, Johnson-Stoklossa C, Kemp A, Kirk S, Kuk J, Langlois MF, Lear S, McInnes A, Macklin D, Naji L, Manjoo P, Morin MP, Nerenberg K, Patton I, Pedersen S, Pereira L, Piccinini-Vallis H, Poddar M, Poirier P, Prud'homme D, Salas XR, Rueda-Clausen C, Russell-Mayhew S, Shiau J, Sherifali D, Sievenpiper J, Sockalingam S, Taylor V, Toth E, Twells L, Tytus R, Walji S, Walker L, Wicklum S (2020). Obesity in adults: a clinical practice guideline. CMAJ.

[4] Breen C, O'Connell J, Geoghegan J, O'Shea D, Birney S, Tully L, Gaynor K, O'Kelly M, O'Malley G, O'Donovan C, Lyons O, Flynn M, Allen S, Arthurs N, Browne S, Byrne M, Callaghan S, Collins C, Courtney A, Crotty M, Donohue C, Donovan C, Dunlevy C, Duggan D, Fearon N, Finucane F, Fitzgerald I, Foy S, Garvey J, Gibson I, Glynn L, Gregg E, Griffin A, Harrington JM, Heary C, Heneghan H, Hogan A, Hynes M, Kearney C, Kelly D, Neff K, le Roux CW, Manning S, McAuliffe F, Moore S, Moran N, Murphy M, Murrin C, O'Brien SM, O'Donnell C, O'Dwyer S, O'Grada C, O'Malley E, O'Reilly O, O'Reilly S, Porter O, Roche HM, Rhynehart A, Ryan L, Seery S, Soare C, Shaamile F, Walsh A, Woods C, Woods C, Yoder R (2022). Obesity in adults: a 2022 adapted clinical practice guideline for Ireland. Obes Facts.

[5] (2014). Obesity: identification, assessment and management. National Institute for Health and Care Excellence.

[6] Tylka TL, Annunziato RA, Burgard D, Daníelsdóttir S, Shuman E, Davis C, Calogero RM (2014). The weight-inclusive versus weight-normative approach to health: evaluating the evidence for prioritizing well-being over weight loss. J Obes.

[7] Barrett S, Begg S, O'Halloran P, Kingsley M (2018). Integrated motivational interviewing and cognitive behaviour therapy for lifestyle mediators of overweight and obesity in community-dwelling adults: a systematic review and meta-analyses. BMC Public Health.

[8] Miller WR, Rollnick S (2002). Motivational Interviewing: Preparing People for Change Second Edition.

[9] Holmes EA, Ghaderi A, Harmer CJ, Ramchandani PG, Cuijpers P, Morrison AP, Roiser JP, Bockting CL, O'Connor RC, Shafran R, Moulds ML, Craske MG (2018). The Lancet Psychiatry Commission on psychological treatments research in tomorrow's science. Lancet Psychiatry.

[10] Kazdin AE (2015). Technology-based interventions and reducing the burdens of mental illness: perspectives and comments on the special series. Cognit Behav Pract.

[11] Wind TR, Rijkeboer M, Andersson G, Riper H (2020). The COVID-19 pandemic: the 'black swan' for mental health care and a turning point for e-health. Internet Interv.

[12] Gutiérrez-Maldonado J, Wiederhold BK, Riva G (2016). Future directions: how virtual reality can further improve the assessment and treatment of eating disorders and obesity. Cyberpsychol Behav Soc Netw.

[13] Castelnuovo G, Simpson S (2011). Ebesity - e-health for obesity - new technologies for the treatment of obesity in clinical psychology and medicine. Clin Pract Epidemiol Ment Health.

[14] Riva G, Gutiérrez-Maldonado J, Wiederhold BK (2016). Virtual worlds versus real body: virtual reality meets eating and weight disorders. Cyberpsychol Behav Soc Netw.

[15] Al-Rasheed A, Alabdulkreem E, Alduailij M, Alduailij M, Alhalabi W, Alharbi S, Lytras MD (2022). Virtual reality in the treatment of patients with overweight and obesity: a systematic review. Sustainability.

[16] Manzoni GM, Cesa GL, Bacchetta M, Castelnuovo G, Conti S, Gaggioli A, Mantovani F, Molinari E, Cárdenas-López G, Riva G (2016). Virtual reality-enhanced cognitive-behavioral therapy for morbid obesity: a randomized controlled study with 1 year follow-up. Cyberpsychol Behav Soc Netw.

[17] Navarro J, Cebolla A, Llorens R, Borrego A, Baños RM (2020). Manipulating self-avatar body dimensions in virtual worlds to complement an internet-delivered intervention to increase physical activity in overweight women. Int J Environ Res Public Health.

[18] Thomas JG, Goldstein CM, Bond DS, Hadley W, Tuerk PW (2020). Web-based virtual reality to enhance behavioural skills training and weight loss in a commercial online weight management programme: the experience success randomized trial. Obes Sci Pract.

[19] Phelan SM, Burgess DJ, Yeazel MW, Hellerstedt WL, Griffin JM, van Ryn M (2015). Impact of weight bias and stigma on quality of care and outcomes for patients with obesity. Obes Rev.

[20] Annesi JJ, Powell SM (2024). The role of change in self-efficacy in maintaining exercise-associated improvements in mood beyond the initial 6 months of expected weight loss in women with obesity. Int J Behav Med.

[21] Botvinick M, Cohen J (1998). Rubber hands 'feel' touch that eyes see. Nature.

[22] Slater M, Spanlang B, Sanchez-Vives MV, Blanke O (2010). First person experience of body transfer in virtual reality. PLoS One.

[23] Petkova VI, Ehrsson HH (2008). If I were you: perceptual illusion of body swapping. PLoS One.

[24] Osimo SA, Pizarro R, Spanlang B, Slater M (2015). Conversations between self and self as Sigmund Freud--a virtual body ownership paradigm for self counselling. Sci Rep.

[25] Slater M, Neyret S, Johnston T, Iruretagoyena G, Crespo MÁ, Alabèrnia-Segura M, Spanlang B, Feixas G (2019). An experimental study of a virtual reality counselling paradigm using embodied self-dialogue. Sci Rep.

[26] Anastasiadou D, Slater M, Spanlang B, Cano Porras D, Comas M, Ciudin A, Puig GP, Vázquez-De Sebastián J, Ramos-Quiroga JA, Lusilla-Palacios P (2022). Clinical efficacy of a virtual reality tool for the treatment of obesity: study protocol of a randomised controlled trial. BMJ Open.

[27] Anastasiadou D, Herrero P, Vázquez-De Sebastián J, Garcia-Royo P, Spanlang B, Álvarez de la Campa E, Slater M, Ciudin A, Comas M, Ramos-Quiroga JA, Lusilla-Palacios P (2023). Virtual self-conversation using motivational interviewing techniques to promote healthy eating and physical activity: a usability study. Front Psychiatry.

[28] Harris PA, Taylor R, Thielke R, Payne J, Gonzalez N, Conde JG (2009). Research electronic data capture (REDCap)--a metadata-driven methodology and workflow process for providing translational research informatics support. J Biomed Inform.

[29] Harris PA, Taylor R, Minor BL, Elliott V, Fernandez M, O'Neal L, McLeod L, Delacqua G, Delacqua F, Kirby J, Duda SN (2019). The REDCap consortium: building an international community of software platform partners. J Biomed Inform.

[30] Andrés A, Saldaña C, Gómez-Benito J (2011). The transtheoretical model in weight management: validation of the processes of change questionnaire. Obes Facts.

[31] Karlsson J, Persson LO, Sjöström L, Sullivan M (2000). Psychometric properties and factor structure of the Three-Factor Eating Questionnaire (TFEQ) in obese men and women. Results from the Swedish Obese Subjects (SOS) study. Int J Obes Relat Metab Disord.

[32] Jáuregui-Lobera I, García-Cruz P, Carbonero-Carreño R, Magallares A, Ruiz-Prieto I (2014). Psychometric properties of Spanish version of the Three-Factor Eating Questionnaire-R18 (Tfeq-Sp) and its relationship with some eating- and body image-related variables. Nutrients.

[33] Castro Rodríguez P, Bellido Guerrero D, Pertega Díaz S, Grupo Colaborativo del Estudio (2010). [Design and validation of a new dietary habits questionnaire for the overweight and obese]. Endocrinol Nutr.

[34] Zigmond AS, Snaith RP (1983). The hospital anxiety and depression scale. Acta Psychiatr Scand.

[35] Quintana JM, Padierna A, Esteban C, Arostegui I, Bilbao A, Ruiz I (2003). Evaluation of the psychometric characteristics of the Spanish version of the hospital anxiety and depression scale. Acta Psychiatr Scand.

[36] Warren CS, Cepeda-Benito A, Gleaves DH, Moreno S, Rodriguez S, Fernandez MC, Fingeret MC, Pearson CA (2008). English and Spanish versions of the body shape questionnaire: measurement equivalence across ethnicity and clinical status. Int J Eat Disord.

[37] Macho S, Andrés A, Saldaña C (2021). Validation of the modified weight bias internalization scale in a Spanish adult population. Clin Obes.

[38] Rami L, Valls-Pedret C, Bartrés-Faz D, Caprile C, Solé-Padullés C, Castellvi M, Olives J, Bosch B, Molinuevo JL (2011). [Cognitive reserve questionnaire. Scores obtained in a healthy elderly population and in one with Alzheimer's disease]. Rev Neurol.

[39] Gil-Gómez JA, Manzano-Hernández P, Albiol-Pérez S, Aula-Valero C, Gil-Gómez H, Lozano-Quilis JA (2017). USEQ: a short questionnaire for satisfaction evaluation of virtual rehabilitation systems. Sensors (Basel).

[40] Mainzer R, Apajee J, Nguyen CD, Carlin JB, Lee KJ (2021). A comparison of multiple imputation strategies for handling missing data in multi-item scales: guidance for longitudinal studies. Stat Med.

[41] Eekhout I, de Vet HC, de Boer MR, Twisk JW, Heymans MW (2018). Passive imputation and parcel summaries are both valid to handle missing items in studies with many multi-item scales. Stat Methods Med Res.

[42] Zettle RD, Rains JC, Hayes SC (2011). Processes of change in acceptance and commitment therapy and cognitive therapy for depression: a mediation reanalysis of Zettle and Rains. Behav Modif.

[43] Nakagawa S, Johnson PC, Schielzeth H (2017). The coefficient of determination and intra-class correlation coefficient from generalized linear mixed-effects models revisited and expanded. J R Soc Interface.

[44] Nakagawa S, Schielzeth H (2012). A general and simple method for obtaining R2 from generalized linear mixed-effects models. Methods Ecol Evol.

[45] Cohen J (1988). Statistical Power Analysis for the Behavioral Sciences.

[46] van Buuren S, Groothuis-Oudshoorn K (2011). mice: multivariate imputation by chained equations in R. J Stat Softw.

[47] Tod D, Hardy J, Oliver E (2011). Effects of self-talk: a systematic review. J Sport Exerc Psychol.

[48] Bergh I, Lundin Kvalem I, Risstad H, Sniehotta FF (2016). Preoperative predictors of adherence to dietary and physical activity recommendations and weight loss one year after surgery. Surg Obes Relat Dis.

[49] Cesa GL, Manzoni GM, Bacchetta M, Castelnuovo G, Conti S, Gaggioli A, Mantovani F, Molinari E, Cárdenas-López G, Riva G (2013). Virtual reality for enhancing the cognitive behavioral treatment of obesity with binge eating disorder: randomized controlled study with one-year follow-up. J Med Internet Res.

[50] Pyykkö JE, Aydin Ö, Gerdes VE, Acherman YI, Groen AK, van de Laar AW, Nieuwdorp M, Sanderman R, Hagedoorn M (2022). Psychological functioning and well-being before and after bariatric surgery; what is the benefit of being self-compassionate?. Br J Health Psychol.

[51] Johnston JD, Massey AP, Devaneaux CA (2012). Innovation in weight loss programs: a 3-dimensional virtual-world approach. J Med Internet Res.

[52] Andreu A, Flores L, Molero J, Mestre C, Obach A, Torres F, Moizé V, Vidal J, Navinés R, Peri JM, Cañizares S (2022). Patients undergoing bariatric surgery: a special risk group for lifestyle, emotional and behavioral adaptations during the COVID-19 lockdown. Lessons from the first wave. Obes Surg.

[53] Lecube A, Sánchez E, Andrés A, Saldaña C, Morales MJ, Calañas A, Miñambres I, Pellitero S, Cordido F, Bueno M, Caixàs A, Vilarrasa N (2019). Assessing motivational stages and processes of change for weight management around bariatric surgery: a multicenter study. Obes Surg.

[54] Wee CC, Huskey KW, Bolcic-Jankovic D, Colten ME, Davis RB, Hamel M (2014). Sex, race, and consideration of bariatric surgery among primary care patients with moderate to severe obesity. J Gen Intern Med.

[55] Mousapour P, Tasdighi E, Khalaj A, Mahdavi M, Valizadeh M, Taheri H, Hosseinpanah F, Barzin M (2021). Sex disparity in laparoscopic bariatric surgery outcomes: a matched-pair cohort analysis. Sci Rep.

[56] Walędziak M, Różańska-Walędziak A, Pędziwiatr M, Szeliga J, Proczko-Stepaniak M, Wysocki M, Stefura T, Major P (2020). Bariatric surgery during COVID-19 pandemic from patients' point of view-the results of a national survey. J Clin Med.

[57] Bjelland I, Dahl AA, Haug TT, Neckelmann D (2002). The validity of the hospital anxiety and depression scale. An updated literature review. J Psychosom Res.

[58] Barbuti M, Brancati GE, Calderone A, Fierabracci P, Salvetti G, Weiss F, Carignani G, Santini F, Perugi G (2022). Prevalence of mood, panic and eating disorders in obese patients referred to bariatric surgery: patterns of comorbidity and relationship with body mass index. Eat Weight Disord.

[59] Usubini AG, Cattivelli R, Villa V, Varallo G, Granese V, Pietrabissa G, Manzoni GM, Castelnuovo G, Molinari E, Saiz-Sapena N, Oviedo JM (2020). Psychological considerations for bariatric surgery. Bariatric Surgery - From the Non-Surgical Approach to the Post-Surgery Individual Care.

[60] Cohn I, Raman J, Sui Z (2019). Patient motivations and expectations prior to bariatric surgery: a qualitative systematic review. Obes Rev.

[61] Ahlich E, Verzijl CL, Cunning A, Wright E, Rancourt D (2021). Patient motivations and goals for bariatric surgery: a mixed methods study. Surg Obes Relat Dis.

[62] Annesi JJ, Gorjala S (2010). Relations of self-regulation and self-efficacy for exercise and eating and BMI change: a field investigation. Biopsychosoc Med.

[63] Riva G, Bacchetta M, Baruffi M, Molinari E (2001). Virtual reality-based multidimensional therapy for the treatment of body image disturbances in obesity: a controlled study. Cyberpsychol Behav.

[64] Manzoni GM, Pagnini F, Gorini A, Preziosa A, Castelnuovo G, Molinari E, Riva G (2009). Can relaxation training reduce emotional eating in women with obesity? An exploratory study with 3 months of follow-up. J Am Diet Assoc.

[65] Manzoni GM, Gorini A, Preziosa A, Pagnini F, Castelnuovo G, Molinari E, Riva G (2008). New technologies and relaxation: an explorative study on obese patients with emotional eating. J Cyberther Rehabil.

[66] Freeman D, Reeve S, Robinson A, Ehlers A, Clark D, Spanlang B, Slater M (2017). Virtual reality in the assessment, understanding, and treatment of mental health disorders. Psychol Med.

