# Supplementary figures and images for: Assessing the Clinical Efficacy of a Virtual Reality Tool for the Treatment of Obesity: Randomized Controlled Trial
Source: J Med Internet Res. 2024 Apr 5;26:e51558. doi: 10.2196/51558 (PMC11031704; doi:10.2196/51558)

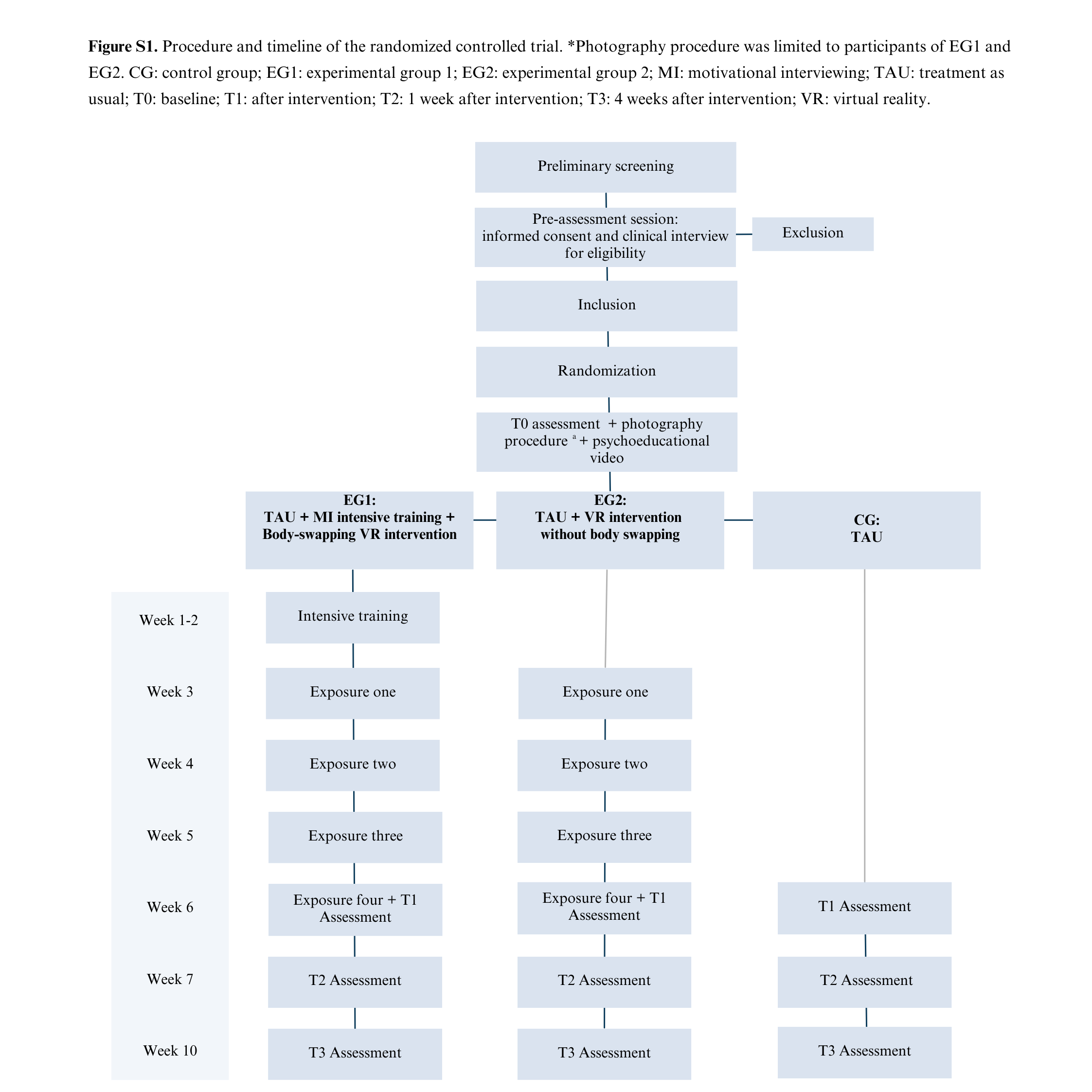

Supplement: Multimedia Appendix 1 [file jmir_v26i1e51558_app1.png]

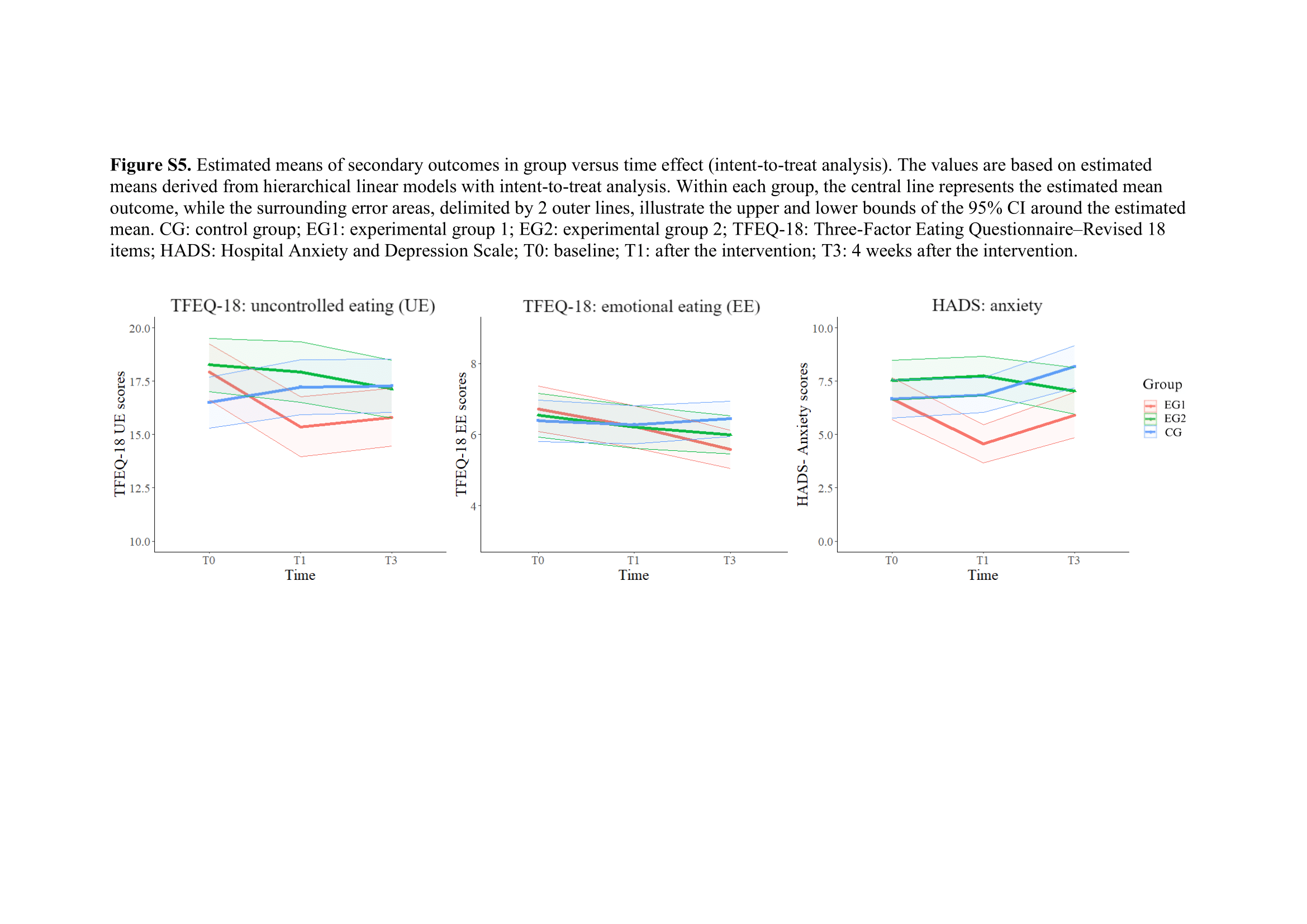

Supplement: Multimedia Appendix 5 [file jmir_v26i1e51558_app5.png]
