# Supplementary material for: Assessing the Clinical Efficacy of a Virtual Reality Tool for the Treatment of Obesity: Randomized Controlled Trial
Source: J Med Internet Res. 2024 Apr 5;26:e51558. doi: 10.2196/51558 (PMC11031704; doi:10.2196/51558)
Supplement: Multimedia Appendix 2 [file jmir_v26i1e51558_app2.docx]

Table S1. Sociodemographic and clinical characteristics of all participants, and separately for each group.

|  | | |  |  | Total (N=68) | Experimental group 1  (n=24) | Experimental group 2  (n=22) | Control group  (n=22) | *P* value |
| --- | --- | --- | --- | --- | --- | --- | --- | --- | --- |
|  | | |  |  |  |  |  |  |  |
| **Age (years), mean (SD)** | | |  |  | 44.22 (10.3) | 46 (10.58) | 44.05 (10.53) | 42.46 (9.9) | .65 |
| **Sex, n (%)** | | |  |  |  |  |  |  | .02 |
|  | Female | | | | 54 (79.4) | 22 (91.7) | 13 (59.1) | 19 (86.4) |  |
|  | Male | | | | 14 (20.6) | 2 (8.3) | 9 (40.9) | 3 (13.6) |  |
| **Nationality, n (%)** | | |  |  |  |  |  |  | .15 |
|  | Spanish | | | | 51 (75) | 20 (83.3) | 17 (77.3) | 14 (63.6) |  |
|  | Latin-American | | | | 11 (16.2) | 2 (8.3) | 5 (22.7) | 4 (18.2) |  |
|  | Latin-American and Spanish | | | | 5 (7.35) | 1 (4.2) |  | 4 (18.2) |  |
|  | African | | | | 1 (1.5) | 1 (4.2) |  |  |  |
| **Employment status,**  **n (%)** | | |  |  |  |  |  |  | .45 |
|  | Full-time job | | | | 29 (42.6) | 7 (29.2) | 9 (40.9) | 13 (59.1) |  |
|  | Unemployed | | | | 16 (23.5) | 5 (20.8) | 7 (31.8) | 4 (18.2) |  |
|  | Part-time job | | | | 10 (14.7) | 5 (20.8) | 3 (13.6) | 2 (9.1) |  |
|  | Retired | | | | 5 (7.4) | 3 (12.5) | 1 (4.5) | 1 (4.5) |  |
|  | Sick leave | | | | 4 (5.9) | 3 (12.5) |  | 1 (4.5) |  |
|  | Student | | | | 2 (2.9) | 1 (4.2) | 1 (4.5) |  |  |
|  | Housework | | | | 1 (1.5) |  | 1 (4.5) |  |  |
| **Highest level of education, n (%)** | | |  |  |  |  |  |  | .56 |
|  | Uneducated | | | | 1 (1.5) |  | 1 (4.5) |  |  |
|  | Elementary school | | | | 5 (7.4) |  | 2 (9.1) | 3 (13.6) |  |
|  | High school | | | | 30 (44.1) | 10 (41.7) | 10 (45.5) | 10 (45.5) |  |
|  | Vocational training | | | | 22 (32.4) | 8 (33.3) | 7 (31.8) | 7 (31.8) |  |
|  | Undergraduate | | | | 7 (10.3) | 4 (16.7) | 1 (4.5) | 2 (9.1) |  |
|  | Postgraduate | | | | 3 (4.4) | 2 (8.3) | 1 (4.5) |  |  |
| **Marital status, n (%)** | | |  |  |  |  |  |  | .96 |
|  | Single | | | | 21 (30.9) | 6 (25) | 7 (31.8) | 8 (36.4) |  |
|  | Married | | | | 34 (50) | 12 (50) | 11 (50) | 11 (50) |  |
|  | Domestic partnership | | | | 7 (10.3) | 3 (12.5) | 2 (9.1) | 2 (9.1) |  |
|  | Divorced/Separated | | | | 6 (8.8) | 3 (12.5) | 2 (9.1) | 1 (4.5) |  |
| **Currently living together, n (%)** | | | | |  |  |  |  | .09 |
|  | Living alone | | | | 4 (5.9) | 1 (4.2) | 2 (9.1) | 1 (4.5) |  |
|  | Significant person (ex: friend) | | | | 1 (1.5) | 1 (4.2) |  |  |  |
|  | Partner | | | | 13 (19.1) | 9 (37.5) | 2 (9.1) | 2 (9.1) |  |
|  | Family | | | | 50 (73.5) | 13 (54.2) | 18 (81.8) | 19 (86.4) |  |
| **Clinical data** | | |  |  |  |  |  |  |  |
| **BMI, mean (SD)** | | |  |  | 43.58 (5.96) | 43.44 (4.94) | 42.82 (6.83) | 44.5 (6.2) | .51 |
| **Treatment duration (months), mean (SD) ^a^** | | |  |  | 21.62 (11.18) | 21.86 (10.74) | 23 (12.02) | 10 (11.04) | .67 |
| **Antecedents of mental illness, n (%)** | | |  |  |  |  |  |  | .66 |
|  | Yes |  | | | 32 (47.1) | 13 (45.8) | 10 (45.5) | 9 (40.9) |  |
|  | No |  | | | 36 (52.9) | 11 (54.2) | 12 (54.5) | 13 (59.1) |  |
| **Current mental illness, n (%/%^b^)** | | |  |  |  |  |  |  | .02 |
|  | Yes |  | | | 15 (22.1) | 10 (41.7) | 2 (9.1) | 3 (13.6) |  |
|  |  | Anxiety | | | 8 (53.3/11.7) | 5 (50/20.8) | 2 (100/9.1) | 1 (33.3/4.5) |  |
|  |  | Depression | | | 6 (40/8.8) | 4 (40/16.7) | 1 (50/4.5) | 1 (33.3/4.5) |  |
|  |  | Adjustment disorder | | | 2 (13.3/2,9) | 2 (20/8.3) |  |  |  |
|  |  | Other (ADHD, bipolar disorder,…) | | | 6 (40/8.8) | 4 (40%16.7) |  | 2 (66.6/9.1) |  |
|  | No |  | | | 53 (77.9) | 14 (58.3) | 20 (90.9) | 19 (86.4) |  |
| **Physical comorbidities, n (%/%^b^)** |  |  | | |  |  |  |  |  |
|  | Yes |  | | | 58 (85.3) | 18 (75) | 18 (81.8) | 22 (100) | .049 |
|  |  | Pain | | | 18 (31/26.4) | 6 (33.3/25) | 4 (22.2/18.2) | 8 (36.4) |  |
|  |  | Breathing problems | | | 15 (25.9/22.1) | 3 (16.7/12.5) | 7 (38.9/31.8) | 5 (22.7) |  |
|  |  | Endocrine problems | | | 16 (27.6/23.5) | 4 (22.2/16.7) | 6 (33.3/27.3) | 6 (27.3) |  |
|  |  | Cardiovascular problems | | | 15 (25.9/22.1) | 6 (33.3/25) | 2 (11.1/9.1) | 7 (31.8) |  |
|  |  | Gastrointestinal problems | | | 3 (5.2/4.4) | 1 (5.5/4.2) |  | 2 (9.1) |  |
|  |  | Neurological problems | | | 8 (13.8/11.8) | 1 (5.5/4.2) | 4 (22.2/18.2) | 3 (13.6) |  |
|  |  | Other problems (skin, kidney problems,…) | | | 9 (15.5/13.23) | 4 (22.2/16.7) | 4 (22.2/18.2) | 4 (18.2) |  |
|  | No |  | | | 10 (14.7) | 6 (25) | 4 (18.2) |  |  |

Note: ^a^ N=66 for treatment duration due to two missing values. ^b^ the first percentage corresponds to those who replied “yes” and the second percentage to those from the total sample.

Abbreviation: BMI, body mass index; ADHD, Attention deficit hyperactivity disorder.
