# Supplementary material for: Assessing the Clinical Efficacy of a Virtual Reality Tool for the Treatment of Obesity: Randomized Controlled Trial
Source: J Med Internet Res. 2024 Apr 5;26:e51558. doi: 10.2196/51558 (PMC11031704; doi:10.2196/51558)
Supplement: Multimedia Appendix 3 [file jmir_v26i1e51558_app3.docx]

Table S2. Descriptive statistics (mean, SD) on scales and subscales of the study divided into groups and time measures.

|  | | T0^a^ | | | | | | T1^b^ | | | | | | T2^c^ | | | | | | T3^d^ | | | | | |
| --- | --- | --- | --- | --- | --- | --- | --- | --- | --- | --- | --- | --- | --- | --- | --- | --- | --- | --- | --- | --- | --- | --- | --- | --- | --- |
|  | | EG1^e^ | | EG2^f^ | | GC^g^ | | EG1 | | EG2 |  | CG |  | EG1 |  | EG2 |  | CG |  | EG1 |  | EG2 |  | CG |  |
|  |  | N | Mean (SD) | N | Mean (SD) | N | Mean (SD) | N | Mean (SD) | N | Mean (SD) | N | Mean (SD) | N | Mean (SD) | N | Mean (SD) | N | Mean (SD) | N | Mean (SD) | N | Mean (SD) | N | Mean (SD) |
| **RR**^h^ | |  |  |  |  |  |  |  |  |  |  |  |  |  |  |  |  |  |  |  |  |  |  |  |  |
|  | RR: importance weight | 24 | 8.96  (1.33) | 22 | 8.73  (1.83) | 22 | 9.09  (1.11) | 18 | 9.5  (.79) | 14 | 9.36  (.93) | 20 | 9.2  (1.00) | 16 | 9.50  (0.82) | 12 | 9.25  (1.14) | 16 | 9.25  (1.13) | 16 | 9.38  (.89) | 13 | 9.38  (.87) | 16 | 9.31  (0.87) |
|  | RR: importance exercise | 24 | 8  (2.11) | 22 | 7.45  (1.90) | 22 | 8  (1.23) | 18 | 9  (1.68) | 14 | 8.29  (1.14) | 20 | 8.35  (1.53) | 16 | 9  (1.37) | 12 | 7.75  (1.36) | 16 | 8.56  (1.5) | 16 | 8.94  (1.61) | 13 | 8.23  (1.36) | 16 | 8.31  (1.35) |
|  | RR: confidence weight | 24 | 8  (2.11) | 22 | 7.77  (2.00) | 22 | 7.91  (1.77) | 18 | 8.67  (1.24) | 14 | 8.64  (1.5) | 20 | 8.05  (1.64) | 16 | 8.56  (1.75) | 12 | 8.83  (1.19) | 16 | 8.19  (1.68) | 16 | 8.5  (1.90) | 13 | 8.54  (1.51) | 16 | 8.06  (1.44) |
|  | RR: confidence exercise | 24 | 7.38  (2.10) | 22 | 6.68  (1.89) | 22 | 7.14  (1.75 | 18 | 8.11  (1.86) | 14 | 7.64  (1.7) | 20 | 7.65  (1.60) | 16 | 8.31  (1.70) | 12 | 7.33  (1.83) | 16 | 7.31  (1.66) | 16 | 8.25  (1.92) | 13 | 7.23  (1.69) | 16 | 7.94  (1.77) |
|  | RR: preparation weight | 24 | 7.96  (1.92) | 22 | 7.82  (2.40) | 22 | 8.18  (1.62) | 18 | 8.83  (1.38) | 14 | 9.21  (.98) | 20 | 8.9  (1.59) | 16 | 8.75  (1.73) | 12 | 8.92  (1.24) | 16 | 8.31  (1.66) | 16 | 8.56  (2) | 13 | 9.08  (.95) | 16 | 8.56  (1.93) |
|  | RR: preparation exercise | 24 | 7.38  (1.8) | 22 | 6.73  (1.75) | 22 | 7.18  (1.53) | 18 | 8.28  (1.93) | 14 | 7.57  (1.51) | 20 | 7.6  (1.73) | 16 | 8.56  (1.71) | 12 | 7.67  (1.44) | 16 | 7.31  (1.82) | 16 | 8.44  (2) | 13 | 7.85  (1.28) | 16 | 7.75  (1.73) |
| **Eating Habits** | |  |  |  |  |  |  |  |  |  |  |  |  |  |  |  |  |  |  |  |  |  |  |  |  |
|  | HABITS: total score | 23 | 3.53  (0.49) | 22 | 3.46  (.55) | 21 | 3.34  (.43) | 16 | 3.68  (0.44) | 14 | 3.69  (.41) | 19 | 3.56  (.39) |  |  |  |  |  |  | 15 | 3.83  (.48) | 13 | 3.70  (.43) | 17 | 3.67  (.37) |
| **HADS**^i^ | |  |  |  |  |  |  |  |  |  |  |  |  |  |  |  |  |  |  |  |  |  |  |  |  |
|  | HADS: depression | 23 | 7.43  (4.36) | 22 | 6.04  (3.5) | 22 | 5.27  (3.55) | 17 | 5  (3.35) | 14 | 3.93  (2.73) | 19 | 4.11  (2.88) |  |  |  |  |  |  | 15 | 6.07  (4.86) | 13 | 4.23  (2.68) | 17 | 5.82  (4.38) |
|  | HADS: anxiety | 23 | 8.52  (4.50) | 22 | 7.14  (4.57) | 22 | 6.68  (3.73) | 17 | 5.59  (2.94) | 14 | 6  (4.13) | 19 | 6.63  (3.7) |  |  |  |  |  |  | 15 | 7.13  (4.56) | 13 | 5.23  (3.44) | 17 | 8.65  (5.11) |
| **P-Weight**^j^ **& S-Weight**^k^ | |  |  |  |  |  |  |  |  |  |  |  |  |  |  |  |  |  |  |  |  |  |  |  |  |
|  | P-Weight: REm^l^ | 24 | 54.1  (6.42) | 22 | 54.41  (5.89) | 22 | 54.09  (6.54) | 17 | 50.65  (6.67) | 14 | 51.86  (8.18) | 20 | 52.2  (10.3) |  |  |  |  |  |  | 16 | 52.63  (7.55) | 13 | 51.23  (7.21) | 17 | 54.35  (7.63) |
|  | P-Weight: ACP^m^ | 24 | 23.67  (5.66) | 22 | 24.73  (4.14) | 22 | 24.5  (4.21) | 17 | 24.35  (5.14) | 14 | 25.14  (4.2) | 20 | 24.65  (4.78) |  |  |  |  |  |  | 16 | 24.69  (6.73) | 13 | 24.08  (5.42) | 17 | 26.59  (4.08) |
|  | P-Weight: REn^n^ | 24 | 14.41  (5.17) | 22 | 14.91  (4.11) | 22 | 14.86  (4.72) | 17 | 14.53  (5.01) | 14 | 15.14  (4.57) | 20 | 15.25  (5.54) |  |  |  |  |  |  | 16 | 15.5  (4.89) | 13 | 13.92  (3.73) | 17 | 14.88  (4.86) |
|  | P-Weight: ECP^o^ | 24 | 33.75  (6.92) | 22 | 34.45  (5.36) | 22 | 33.14  (6.77) | 17 | 32.59  (5.67) | 14 | 33.21  (5.28) | 20 | 32.4  (7.07) |  |  |  |  |  |  | 16 | 35  (6.64) | 13 | 32.38  (4.93) | 17 | 33.82  (7.92) |
|  | S-Weight | 24 | 4.33  (0.92) | 20 | 4.70  (0.57) | 22 | 4.36  (0.66) | 17 | 4.53  (0.62) | 12 | 4.83  (0.39) | 19 | 4.63  (0.76) |  |  |  |  |  |  | 15 | 4.27  (0.80) | 12 | 4.75  (.45) | 16 | 4.75  (.45) |
| **TFEQ-R18**^p^ | |  |  |  |  |  |  |  |  |  |  |  |  |  |  |  |  |  |  |  |  |  |  |  |  |
|  | TFEQ-R18: UE^q^ | 24 | 18.92  (6.35) | 22 | 19.32  (6.42) | 22 | 16.09  (3.93) | 17 | 16  (4.61) | 14 | 17.14  (4.62) | 19 | 16.68  (5.35) |  |  |  |  |  |  | 16 | 16.44  (4.73) | 13 | 16.23  (4.62) | 17 | 16.76  (4.83) |
|  | TFEQ-R18: EE^r^ | 24 | 6.88  (2.72) | 22 | 7  (2.88) | 22 | 6.09  (2.18) | 17 | 6.59  (2.72) | 14 | 6  (1.57) | 19 | 6.05  (2.09) |  |  |  |  |  |  | 16 | 5.88  (2.39) | 13 | 5.77  (1.74) | 17 | 6.12  (1.83) |
|  | TFEQ-R18: CR^s^ | 24 | 16.25  (3.80) | 22 | 15.55  (3.50) | 22 | 15.73  (2.1) | 17 | 16.29  (3.37) | 14 | 17.64  (2.65) | 19 | 16.42  (2.12) |  |  |  |  |  |  | 16 | 17.5  (3.61) | 13 | 17.62  (2.57) | 17 | 17.41  (2.6) |
| **BSQ-10**^t^ | |  |  |  |  |  |  |  |  |  |  |  |  |  |  |  |  |  |  |  |  |  |  |  |  |
|  | BSQ-10: total score | 23 | 32.13  (11.29) | 22 | 34.22  (12.9) | 22 |  | 17 | 28.59  (7.24) | 14 | 27.86  (9.94) | 19 | 31.63  (13.06) |  |  |  |  |  |  | 16 | 31.87  (9.92) | 13 | 29.61  (11.53) | 17 | 36.71  (14.26) |
| **WBIS-M**^u^ | |  |  |  |  |  |  |  |  |  |  |  |  |  |  |  |  |  |  |  |  |  |  |  |  |
|  | WBIS-M: Total score | 23 | 4.05  (1.07) | 22 | 4.6  (1.37) | 22 | 4.1  (1.41) | 17 | 4.01  .99 | 14 | 3.71  (1.15) | 19 | 3.78  (1.07) |  |  |  |  |  |  | 16 | 4.23  1.12 | 13 | 3.83  (1.49) | 17 | 4.08  (1.27) |

^a^ T0: baseline.

^b^ T1: after the intervention.

^c^ T2: 1 week after the intervention.

^d^ T3: 4 weeks after the intervention.

^e^ EG1: experimental group 1.

^f^ EG2: experimental group 2.

^g^ CG: control group.

^h^ RR: readiness ruler.

^i^ HADS: Hospital Anxiety and Depression Scale.

^j^ P-Weight: Processes of Change Questionnaire for Weight Management.

^k^ S-Weight: Stages of Change Questionnaire for Weight Management.

^l^ REm: emotional reevaluation.

^m^ ACP: weight consequences evaluation.

^n^ REn: supporting relationships.

^o^ ECP: weight management actions.

^p^ TFEQ-R18: Three-Factor Eating Questionnaire–Revised 18 items.

^q^ UE: uncontrolled eating.

^r^ EE: emotional eating.

^s^ CR: cognitive restrain.

^t^ BSQ-10: Body Shape Questionnaire, 10-item version.

^u^ WBIS-M: Modified Weight Bias Internalization Scale.
