# Supplementary material for: Assessing the Clinical Efficacy of a Virtual Reality Tool for the Treatment of Obesity: Randomized Controlled Trial
Source: J Med Internet Res. 2024 Apr 5;26:e51558. doi: 10.2196/51558 (PMC11031704; doi:10.2196/51558)
Supplement: Multimedia Appendix 4 [file jmir_v26i1e51558_app4.docx]

| Table S3. Stages of Change Questionnaires for Weight Management frequencies and percentages divided into time and groups. | | | | | |  |
| --- | --- | --- | --- | --- | --- | --- |
| Treatment point | | | |  | Question | n (%) |
| **T0^a^** | | | |  |  |  |
|  | EG1^b^ | | | | At the moment I’m not doing anything to lose weight but I’m thinking about doing something over the next 6 months | 1 (6.3) |
|  |  |  |  |  | I’ve been making an effort to lose weight (by dieting and/or exercising) for less than 6 months | 4 (25%) |
|  |  |  |  |  | I’ve been making an effort to maintain my weight (by dieting and/or exercising) for more than 6 months​ | 11 (68.8) |
|  | EG2^c^ | | | | I’ve been making an effort to lose weight (by dieting and/or exercising) for less than 6 months | 1 (8.3) |
|  |  |  |  |  | I’ve been making an effort to maintain my weight (by dieting and/or exercising) for more than 6 months​ | 11 (91.7) |
|  | CG^d^ | | | | I’ve been making an effort to lose weight (by dieting and/or exercising) for less than 6 months | 6 (37.5) |
|  |  |  |  |  | I’ve been making an effort to maintain my weight (by dieting and/or exercising) for more than 6 months​ | 10 (62.5) |
| **T1^e^** | | |  | |  |  |
|  | EG1 | | | | During the last year I haven’t done anything to lose weight but I’m planning to do something over the next 30 days | 1 (6.3) |
|  |  |  |  |  | I’ve been making an effort to lose weight (by dieting and/or exercising) for less than 6 months | 6 (37.5) |
|  |  |  |  |  | I’ve been making an effort to maintain my weight (by dieting1 and/or exercising2) for more than 6 months​ | 9 (56.3) |
|  | EG2 | | | | I’ve been making an effort to lose weight (by dieting and/or exercising) for less than 6 months | 2 (18.2) |
|  |  |  |  |  | I’ve been making an effort to maintain my weight (by dieting and/or exercising) for more than 6 months​ | 9 (81.8) |
|  | CG | | | | I’ve been making an effort to lose weight (by dieting and/or exercising) for less than 6 months | 4 (25) |
|  |  |  |  |  | I’ve been making an effort to maintain my weight (by dieting and/or exercising) for more than 6 months​ | 12 (75) |
| **T3^f^** | |  | | |  |  |
|  | EG1 | | | | At the moment I’m not doing anything to lose weight but I’m thinking about doing something over the next 6 months | 1 (6.7) |
|  |  |  |  |  | I’ve been making an effort to lose weight (by dieting and/or exercising) for less than 6 months | 8 (53.3) |
|  |  |  |  |  | I’ve been making an effort to maintain my weight (by dieting and/or exercising) for more than 6 months​ | 6 (40) |
|  | EG2 | | | | I’ve been making an effort to lose weight (by dieting and/or exercising) for less than 6 months | 3 (25) |
|  |  |  |  |  | I’ve been making an effort to maintain my weight (by dieting and/or exercising) for more than 6 months​ | 9 (75) |
|  | CG | | | | I’ve been making an effort to lose weight (by dieting and/or exercising) for less than 6 months | 4 (25) |
|  |  |  |  |  | I’ve been making an effort to maintain my weight (by dieting and/or exercising) for more than 6 months​ | 12 (75) |

^a^ T0: baseline

^b^ EG1: experimental group 1

^c^ EG2: experimental group 2

^d^ CG: control group

^e^ T1: after the intervention

^f^ T3: 4 weeks after the intervention
