# Supplementary material for: Assessing the Clinical Efficacy of a Virtual Reality Tool for the Treatment of Obesity: Randomized Controlled Trial
Source: J Med Internet Res. 2024 Apr 5;26:e51558. doi: 10.2196/51558 (PMC11031704; doi:10.2196/51558)
Supplement: Multimedia Appendix 6 [file jmir_v26i1e51558_app6.pdf]

CONSORT-EHEALTH (V 1.6.1) - Submission/Publication Form

The CONSORT-EHEALTH checklist is intended for authors of randomized trials evaluating web-based and Internet-based applications/interventions, including mobile interventions, electronic games (incl multiplayer games), social media, certain telehealth applications, and other interactive and/or networked electronic applications. Some of the items (e.g. all subitems under item 5 - description of the intervention) may also be applicable for other study designs.

The goal of the CONSORT EHEALTH checklist and guideline is to be
a) a guide for reporting for authors of RCTs,
b) to form a basis for appraisal of an ehealth trial (in terms of validity)

CONSORT-EHEALTH items/subitems are MANDATORY reporting items for studies published in the Journal of Medical Internet Research and other journals / scientific societies endorsing the checklist.

Items numbered 1., 2., 3., 4a., 4b etc are original CONSORT or CONSORT-NPT (non-pharmacologic treatment) items.
Items with Roman numerals (i., ii, iii, iv etc.) are CONSORT-EHEALTH extensions/clarifications.

As the CONSORT-EHEALTH checklist is still considered in a formative stage, we would ask that you also RATE ON A SCALE OF 1-5 how important/useful you feel each item is FOR THE PURPOSE OF THE CHECKLIST and reporting guideline (optional).

Mandatory reporting items are marked with a red *.
In the textboxes, either copy & paste the relevant sections from your manuscript into this form - please include any quotes from your manuscript in QUOTATION MARKS,
or answer directly by providing additional information not in the manuscript, or elaborating on why the item was not relevant for this study.

YOUR ANSWERS WILL BE PUBLISHED AS A SUPPLEMENTARY FILE TO YOUR PUBLICATION IN JMIR AND ARE CONSIDERED PART OF YOUR PUBLICATION (IF ACCEPTED).
Please fill in these questions diligently. Information will not be copyedited, so please use proper spelling and grammar, use correct capitalization, and avoid abbreviations.

DO NOT FORGET TO SAVE AS PDF _AND_ CLICK THE SUBMIT BUTTON SO YOUR ANSWERS ARE IN OUR DATABASE !!!

Citation Suggestion (if you append the pdf as Appendix we suggest to cite this paper in the caption):
Eysenbach G, CONSORT-EHEALTH Group
CONSORT-EHEALTH: Improving and Standardizing Evaluation Reports of Web-based and Mobile Health Interventions
J Med Internet Res 2011;13(4):e126
URL: http://www.jmir.org/2011/4/e126/
doi: 10.2196/jmir.1923
PMID: 22209829

pherrerocastillo@gmail.com Cambiar de cuenta
No compartido
Borrador guardado

* Indica que la pregunta es obligatoria

Your name *
First Last

Anastasiadou Dimitra

Primary Affiliation (short), City, Country *
University of Toronto, Toronto, Canada

Universitat Autònoma de Barcelona, Barcelona

Your e-mail address *
abc@gmail.com

dimitra.anastasiadou@uab.cat

Title of your manuscript *
Provide the (draft) title of your manuscript.

Assessing the Clinical Efficacy of a Virtual Reality Tool for the Treatment of Obesity: Randomized Controlled Trial

Name of your App/Software/Intervention *
If there is a short and a long/alternate name, write the short name first and add the long name in brackets.

ConVRself

Evaluated Version (if any)
e.g. "V1", "Release 2017-03-01", "Version 2.0.27913"

Tu respuesta

Language(s) *
What language is the intervention/app in? If multiple languages are available, separate by comma (e.g. "English, French")

Spanish

URL of your Intervention Website or App
e.g. a direct link to the mobile app on app in appstore (itunes, Google Play), or URL of the website. If the intervention is a DVD or hardware, you can also link to an Amazon page.

Tu respuesta

URL of an image/screenshot (optional)

Tu respuesta

Accessibility *
Can an enduser access the intervention presently?

- ( ) access is free and open
- (•) access only for special usergroups, not open
- ( ) access is open to everyone, but requires payment/subscription/in-app purchases
- ( ) app/intervention no longer accessible
- ( ) Otro:

Primary Medical Indication/Disease/Condition *
e.g. "Stress", "Diabetes", or define the target group in brackets after the condition, e.g. "Autism (Parents of children with)", "Alzheimers (Informal Caregivers of)"

Obesity

Primary Outcomes measured in trial *
comma-separated list of primary outcomes reported in the trial

Readiness to change habits

Secondary/other outcomes
Are there any other outcomes the intervention is expected to affect?

Lifestyle habits, Eating habits, Psychological Wellbeing, Adherence and satisfaction of VR experiments

Recommended "Dose" *
What do the instructions for users say on how often the app should be used?

- ( ) Approximately Daily
- (•) Approximately Weekly
- ( ) Approximately Monthly
- ( ) Approximately Yearly
- ( ) "as needed"
- ( ) Otro:

Approx. Percentage of Users (starters) still using the app as recommended after 3 months *

- (•) unknown / not evaluated
- ( ) 0-10%
- ( ) 11-20%
- ( ) 21-30%
- ( ) 31-40%
- ( ) 41-50%
- ( ) 51-60%
- ( ) 61-70%
- ( ) 71%-80%
- ( ) 81-90%
- ( ) 91-100%
- ( ) Otro:

Overall, was the app/intervention effective? *

- ( ) yes: all primary outcomes were significantly better in intervention group vs control
- (•) partly: SOME primary outcomes were significantly better in intervention group vs control
- ( ) no statistically significant difference between control and intervention
- ( ) potentially harmful: control was significantly better than intervention in one or more outcomes
- ( ) inconclusive: more research is needed
- ( ) Otro:

Article Preparation Status/Stage *
At which stage in your article preparation are you currently (at the time you fill in this form)

- ( ) not submitted yet - in early draft status
- ( ) not submitted yet - in late draft status, just before submission
- ( ) submitted to a journal but not reviewed yet
- ( ) submitted to a journal and after receiving initial reviewer comments
- (•) submitted to a journal and accepted, but not published yet
- ( ) published
- ( ) Otro:

Journal *
If you already know where you will submit this paper (or if it is already submitted), please provide the journal name (if it is not JMIR, provide the journal name under "other")

- ( ) not submitted yet / unclear where I will submit this
- (•) Journal of Medical Internet Research (JMIR)
- ( ) JMIR mHealth and UHealth
- ( ) JMIR Serious Games
- ( ) JMIR Mental Health
- ( ) JMIR Public Health
- ( ) JMIR Formative Research
- ( ) Other JMIR sister journal
- ( ) Otro:

Is this a full powered effectiveness trial or a pilot/feasibility trial? *

- ( ) Pilot/feasibility
- (•) Fully powered

Manuscript tracking number *
If this is a JMIR submission, please provide the manuscript tracking number under "other" (The ms tracking number can be found in the submission acknowledgement email, or when you login as author in JMIR. If the paper is already published in JMIR, then the ms tracking number is the four-digit number at the end of the DOI, to be found at the bottom of each published article in JMIR)

- (•) no ms number (yet) / not (yet) submitted to / published in JMIR
- ( ) Otro:

TITLE AND ABSTRACT

1a) TITLE: Identification as a randomized trial in the title

1a) Does your paper address CONSORT item 1a? *
I.e does the title contain the phrase "Randomized Controlled Trial"? (if not, explain the reason under "other")

- (•) yes
- ( ) Otro:

1a-i) Identify the mode of delivery in the title
Identify the mode of delivery. Preferably use "web-based" and/or "mobile" and/or "electronic game" in the title. Avoid ambiguous terms like "online", "virtual", "interactive". Use "Internet-based" only if Intervention includes non-web-based Internet components (e.g. email), use "computer-based" or "electronic" only if offline products are used. Use "virtual" only in the context of "virtual reality" (3-D worlds). Use "online" only in the context of "online support groups". Complement or substitute product names with broader terms for the class of products (such as "mobile" or "smart phone" instead of "iphone"), especially if the application runs on different platforms.

subitem not at all important ( ) 1 ( ) 2 ( ) 3 ( ) 4 ( ) 5 essential

Does your paper address subitem 1a-i? *
Copy and paste relevant sections from manuscript title (include quotes in quotation marks "like this" to indicate direct quotes from your manuscript), or elaborate on this item by providing additional information not in the ms, or briefly explain why the item is not applicable/relevant for your study

Assessing the Clinical Efficacy of a Virtual Reality Tool for the Treatment of Obesity: Randomized Controlled Trial

1a-ii) Non-web-based components or important co-interventions in title
Mention non-web-based components or important co-interventions in title, if any (e.g., "with telephone support").

subitem not at all important ( ) 1 ( ) 2 ( ) 3 ( ) 4 ( ) 5 essential

Does your paper address subitem 1a-ii?
Copy and paste relevant sections from manuscript title (include quotes in quotation marks "like this" to indicate direct quotes from your manuscript), or elaborate on this item by providing additional information not in the ms, or briefly explain why the item is not applicable/relevant for your study

No relevant co-interventions to be present in the title.

1a-iii) Primary condition or target group in the title
Mention primary condition or target group in the title, if any (e.g., "for children with Type I Diabetes") Example: A Web-based and Mobile Intervention with Telephone Support for Children with Type I Diabetes: Randomized Controlled Trial

subitem not at all important ( ) 1 ( ) 2 ( ) 3 ( ) 4 ( ) 5 essential

Does your paper address subitem 1a-iii? *
Copy and paste relevant sections from manuscript title (include quotes in quotation marks "like this" to indicate direct quotes from your manuscript), or elaborate on this item by providing additional information not in the ms, or briefly explain why the item is not applicable/relevant for your study

Assessing the Clinical Efficacy of a Virtual Reality Tool for the Treatment of Obesity: Randomized Controlled Trial

1b) ABSTRACT: Structured summary of trial design, methods, results, and conclusions
NPT extension: Description of experimental treatment, comparator, care providers, centers, and blinding status.

1b-i) Key features/functionalities/components of the intervention and comparator in the METHODS section of the ABSTRACT
Mention key features/functionalities/components of the intervention and comparator in the abstract. If possible, also mention theories and principles used for designing the site. Keep in mind the needs of systematic reviewers and indexers by including important synonyms. (Note: Only report in the abstract what the main paper is reporting. If this information is missing from the main body of text, consider adding it)

subitem not at all important ( ) 1 ( ) 2 ( ) 3 ( ) 4 ( ) 5 essential

Does your paper address subitem 1b-i? *
Copy and paste relevant sections from the manuscript abstract (include quotes in quotation marks "like this" to indicate direct quotes from your manuscript), or elaborate on this item by providing additional information not in the ms, or briefly explain why the item is not applicable/relevant for your study

"To tackle these concerns, this study introduces the VR self-counseling paradigm, which incorporates embodiment and body-swapping techniques, along with motivational strategies, to help people living with obesity effectively address some of the root causes of their condition."

1b-ii) Level of human involvement in the METHODS section of the ABSTRACT
Clarify the level of human involvement in the abstract, e.g., use phrases like "fully automated" vs. "therapist/nurse/care provider/physician-assisted" (mention number and expertise of providers involved, if any). (Note: Only report in the abstract what the main paper is reporting. If this information is missing from the main body of text, consider adding it)

subitem not at all important ( ) 1 ( ) 2 ( ) 3 ( ) 4 ( ) 5 essential

Does your paper address subitem 1b-ii?
Copy and paste relevant sections from the manuscript abstract (include quotes in quotation marks "like this" to indicate direct quotes from your manuscript), or elaborate on this item by providing additional information not in the ms, or briefly explain why the item is not applicable/relevant for your study

"In the case of both EG1 and EG2, the VR interventions were assisted by a clinical researcher."

1b-iii) Open vs. closed, web-based (self-assessment) vs. face-to-face assessments in the METHODS section of the ABSTRACT
Mention how participants were recruited (online vs. offline), e.g., from an open access website or from a clinic or a closed online user group (closed usergroup trial), and clarify if this was a purely web-based trial, or there were face-to-face components (as part of the intervention or for assessment). Clearly say if outcomes were self-assessed through questionnaires (as common in web-based trials). Note: In traditional offline trials, an open trial (open-label trial) is a type of clinical trial in which both the researchers and participants know which treatment is being administered. To avoid confusion, use "blinded" or "unblinded" to indicated the level of blinding instead of "open", as "open" in web-based trials usually refers to "open access" (i.e. participants can self-enrol). (Note: Only report in the abstract what the main paper is reporting. If this information is missing from the main body of text, consider adding it)

subitem not at all important ( ) 1 ( ) 2 ( ) 3 ( ) 4 ( ) 5 essential

Does your paper address subitem 1b-iii?
Copy and paste relevant sections from the manuscript abstract (include quotes in quotation marks "like this" to indicate direct quotes from your manuscript), or elaborate on this item by providing additional information not in the ms, or briefly explain why the item is not applicable/relevant for your study

"A randomized controlled trial was conducted with 68 participants from the bariatric surgery waiting list from the obesity unit of the Vall d'Hebron University Hospital in Barcelona, Spain."

1b-iv) RESULTS section in abstract must contain use data
Report number of participants enrolled/assessed in each group, the use/uptake of the intervention (e.g., attrition/adherence metrics, use over time, number of logins etc.), in addition to primary/secondary outcomes. (Note: Only report in the abstract what the main paper is reporting. If this information is missing from the main body of text, consider adding it)

subitem not at all important ( ) 1 ( ) 2 ( ) 3 ( ) 4 ( ) 5 essential

Does your paper address subitem 1b-iv?
Copy and paste relevant sections from the manuscript abstract (include quotes in quotation marks "like this" to indicate direct quotes from your manuscript), or elaborate on this item by providing additional information not in the ms, or briefly explain why the item is not applicable/relevant for your study

"Regarding the primary outcomes, EG1 (24/68, 35%) and EG2 (22/68, 32%) showed significant improvements in confidence to lose weight compared to the CG (22/68, 32%) at all assessment points (β=-.16; P=.02)"

1b-v) CONCLUSIONS/DISCUSSION in abstract for negative trials
Conclusions/Discussions in abstract for negative trials: Discuss the primary outcome - if the trial is negative (primary outcome not changed), and the intervention was not used, discuss whether negative results are attributable to lack of uptake and discuss reasons. (Note: Only report in the abstract what the main paper is reporting. If this information is missing from the main body of text, consider adding it)

subitem not at all important ( ) 1 ( ) 2 ( ) 3 ( ) 4 ( ) 5 essential

Does your paper address subitem 1b-v?
Copy and paste relevant sections from the manuscript abstract (include quotes in quotation marks "like this" to indicate direct quotes from your manuscript), or elaborate on this item by providing additional information not in the ms, or briefly explain why the item is not applicable/relevant for your study

"No significant group versus time effect for the rest of the variables was reported."

INTRODUCTION

2a) In INTRODUCTION: Scientific background and explanation of rationale

2a-i) Problem and the type of system/solution
Describe the problem and the type of system/solution that is object of the study: intended as stand-alone intervention vs. incorporated in broader health care program? Intended for a particular patient population? Goals of the intervention, e.g., being more cost-effective to other interventions, replace or complement other solutions? (Note: Details about the intervention are provided in "Methods" under 5)

subitem not at all important ( ) 1 ( ) 2 ( ) 3 ( ) 4 ( ) 5 essential

Does your paper address subitem 2a-i? *
Copy and paste relevant sections from the manuscript (include quotes in quotation marks "like this" to indicate direct quotes from your manuscript), or elaborate on this item by providing additional information not in the ms, or briefly explain why the item is not applicable/relevant for your study

"While these interventions focus on correcting specific behaviors and provide patients with a safe context to practice eating, emotional, and relational management, they do not adequately address some of the root causes of obesity, such as a lack of motivation to change, low self-efficacy, and the impact of weight stigma interiorization, which are factors that often hinder treatment adherence and long-term maintenance of lifestyle habits"

"To tackle these concerns, this study introduces the VR self-counseling paradigm, which incorporates embodiment and body-swapping techniques, along with motivational strategies, to help people living with obesity effectively address some of the root causes of their condition."

2a-ii) Scientific background, rationale: What is known about the (type of) system
Scientific background, rationale: What is known about the (type of) system that is the object of the study (be sure to discuss the use of similar systems for other conditions/diagnoses, if appropiate), motivation for the study, i.e. what are the reasons for and what is the context for this specific study, from which stakeholder viewpoint is the study performed, potential impact of findings [2]. Briefly justify the choice of the comparator.

subitem not at all important ( ) 1 ( ) 2 ( ) 3 ( ) 4 ( ) 5 essential

Does your paper address subitem 2a-ii? *
Copy and paste relevant sections from the manuscript (include quotes in quotation marks "like this" to indicate direct quotes from your manuscript), or elaborate on this item by providing additional information not in the ms, or briefly explain why the item is not applicable/relevant for your study

"Specifically, the integration of virtual reality (VR) into psychological interventions for these conditions holds great promise in addressing some of the factors associated with the development or maintenance of the disorders. Through the provision of multisensory experiences, extrinsic feedback, and opportunities for embodiment, VR interventions, which are rooted primarily in cognitive behavioral therapy principles, have demonstrated effectiveness as complementary tools in managing obesity and have been associated with promoting behavior changes and weight reduction, reducing binge eating episodes, and addressing body image concerns."

2b) In INTRODUCTION: Specific objectives or hypotheses

Does your paper address CONSORT subitem 2b? *
Copy and paste relevant sections from the manuscript (include quotes in quotation marks "like this" to indicate direct quotes from your manuscript), or elaborate on this item by providing additional information not in the ms, or briefly explain why the item is not applicable/relevant for your study

"The objective of these virtual self-conversations has been to address the following challenges that people living with obesity face: (1) to raise awareness of their actual condition, (2) to better understand and address the impact of weight bias interiorization, and (3) to increase their self-efficacy by setting realistic goals in line with their values."

METHODS

3a) Description of trial design (such as parallel, factorial) including allocation ratio

Does your paper address CONSORT subitem 3a? *
Copy and paste relevant sections from the manuscript (include quotes in quotation marks "like this" to indicate direct quotes from your manuscript), or elaborate on this item by providing additional information not in the ms, or briefly explain why the item is not applicable/relevant for your study

"We conducted an RCT with 3 parallel groups (experimental group 1 [EG1], experimental group 2 [EG2], and a control group [CG]) with a 1:1:1 allocation ratio."

3b) Important changes to methods after trial commencement (such as eligibility criteria), with reasons

Does your paper address CONSORT subitem 3b? *
Copy and paste relevant sections from the manuscript (include quotes in quotation marks "like this" to indicate direct quotes from your manuscript), or elaborate on this item by providing additional information not in the ms, or briefly explain why the item is not applicable/relevant for your study

As stated in the usability study conducted by Anastasiadou et al [27], the BMI criterion was revised to include only a minimum threshold of ≥30 kg/m2 for eligibility to participate in the study, contrary to the BMI criteria originally set in the study protocol (BMI between ≥30 kg/m2 and ≤55 kg/m2) [26].

3b-i) Bug fixes, Downtimes, Content Changes

Bug fixes, Downtimes, Content Changes: ehealth systems are often dynamic systems. A description of changes to methods therefore also includes important changes made on the intervention or comparator during the trial (e.g., major bug fixes or changes in the functionality or content) (5-iii) and other “unexpected events” that may have influenced study design such as staff changes, system failures/downtimes, etc. [2].

	1	2	3	4	5	
subitem not at all important	○	○	○	○	○	essential

Does your paper address subitem 3b-i?

Copy and paste relevant sections from the manuscript (include quotes in quotation marks "like this" to indicate direct quotes from your manuscript), or elaborate on this item by providing additional information not in the ms, or briefly explain why the item is not applicable/relevant for your study

Not reported. There were no unexpected events or bugs to be fixed during the experiments.

4a) Eligibility criteria for participants

Does your paper address CONSORT subitem 4a? *

Copy and paste relevant sections from the manuscript (include quotes in quotation marks "like this" to indicate direct quotes from your manuscript), or elaborate on this item by providing additional information not in the ms, or briefly explain why the item is not applicable/relevant for your study

"To be eligible for inclusion, participants had to meet the following criteria: aged between 18 and 65 years; BMI ≥30 kg/m2; receiving ambulatory treatment at the VHUH; not undergoing any other concurrent treatment specifically related to their obesity condition from other centers; possessing minimal digital skills, which means being able to use a digital device (smartphone, tablet, or computer) to make telephone calls and have video conversations via the internet, send or receive emails, and search for information about products and (health) services; demonstrable oral and written understanding of the Spanish language; and willingness to provide informed consent to participate."

4a-i) Computer / Internet literacy

Computer / Internet literacy is often an implicit “de facto” eligibility criterion - this should be explicitly clarified.

	1	2	3	4	5	
subitem not at all important	○	○	○	○	○	essential

Does your paper address subitem 4a-i?

Copy and paste relevant sections from the manuscript (include quotes in quotation marks "like this" to indicate direct quotes from your manuscript), or elaborate on this item by providing additional information not in the ms, or briefly explain why the item is not applicable/relevant for your study

"possessing minimal digital skills, which means being able to use a digital device (smartphone, tablet, or computer) to make telephone calls and have video conversations via the internet, send or receive emails, and search for information about products and (health) services".

4a-ii) Open vs. closed, web-based vs. face-to-face assessments:

Open vs. closed, web-based vs. face-to-face assessments: Mention how participants were recruited (online vs. offline), e.g., from an open access website or from a clinic, and clarify if this was a purely web-based trial, or there were face-to-face components (as part of the intervention or for assessment), i.e., to what degree got the study team to know the participant. In online-only trials, clarify if participants were quasi-anonymous and whether having multiple identities was possible or whether technical or logistical measures (e.g., cookies, email confirmation, phone calls) were used to detect/prevent these.

	1	2	3	4	5	
subitem not at all important	○	○	○	○	○	essential

Does your paper address subitem 4a-ii? *

Copy and paste relevant sections from the manuscript (include quotes in quotation marks "like this" to indicate direct quotes from your manuscript), or elaborate on this item by providing additional information not in the ms, or briefly explain why the item is not applicable/relevant for your study

"A randomized controlled trial was conducted with 68 participants from the bariatric surgery waiting list from the obesity unit of the Vall d’Hebron University Hospital in Barcelona, Spain."

"Eligible participants were contacted via telephone by a research team member who provided information about the study. If they agreed to participate in the study, an appointment was scheduled at the hospital."

"The data collected at T0, T1, T2, and T3 were obtained through web-based self-report instruments completed by participants using REDCap. In addition, the research team members measured participants’ weight and height to calculate their BMI."

4a-iii) Information giving during recruitment

Information given during recruitment. Specify how participants were briefed for recruitment and in the informed consent procedures (e.g., publish the informed consent documentation as appendix, see also item X26), as this information may have an effect on user self-selection, user expectation and may also bias results.

	1	2	3	4	5	
subitem not at all important	○	○	○	○	○	essential

Does your paper address subitem 4a-iii?

Copy and paste relevant sections from the manuscript (include quotes in quotation marks "like this" to indicate direct quotes from your manuscript), or elaborate on this item by providing additional information not in the ms, or briefly explain why the item is not applicable/relevant for your study

"If they agreed to participate in the study, an appointment was scheduled at the hospital. During this appointment, a clinical interview was conducted by the team member to confirm the participants’ eligibility. Sociodemographic and clinical variables were also collected, and weight and height measurements were taken."

4b) Settings and locations where the data were collected

Does your paper address CONSORT subitem 4b? *

Copy and paste relevant sections from the manuscript (include quotes in quotation marks "like this" to indicate direct quotes from your manuscript), or elaborate on this item by providing additional information not in the ms, or briefly explain why the item is not applicable/relevant for your study

"If they agreed to participate in the study, an appointment was scheduled at the hospital. During this appointment, a clinical interview was conducted by the team member to confirm the participants’ eligibility. Sociodemographic and clinical variables were also collected, and weight and height measurements were taken."

"Two weeks later, EG1 and EG2 participants engaged in 4 VR scenarios at the hospital facilities, assisted by a team clinical researcher. Scenarios were distributed in weekly sessions, each lasting 30 minutes, over a period of 4 weeks."

"Two weeks later, EG1 and EG2 participants engaged in 4 VR scenarios at the hospital facilities, assisted by a team clinical researcher."

4b-i) Report if outcomes were (self-)assessed through online questionnaires

Clearly report if outcomes were (self-)assessed through online questionnaires (as common in web-based trials) or otherwise.

	1	2	3	4	5	
subitem not at all important	○	○	○	○	○	essential

Does your paper address subitem 4b-i? *

Copy and paste relevant sections from the manuscript (include quotes in quotation marks "like this" to indicate direct quotes from your manuscript), or elaborate on this item by providing additional information not in the ms, or briefly explain why the item is not applicable/relevant for your study

"After each exposure, satisfaction and adherence to the VR experiments were assessed for both groups using a semistructured interview designed by the research team. In addition, several self-report questionnaires were administered to the participants, including the readiness ruler (RR), the Suitability Evaluation Questionnaire (SEQ), and the Body Ownership Questionnaire (BOQ; for more detailed information, refer to the Measures subsection)."

"The data collected at T0, T1, T2, and T3 were obtained through web-based self-report instruments completed by participants using REDCap."

4b-ii) Report how institutional affiliations are displayed

Report how institutional affiliations are displayed to potential participants [on ehealth media], as affiliations with prestigious hospitals or universities may affect volunteer rates, use, and reactions with regards to an intervention.(Not a required item – describe only if this may bias results)

	1	2	3	4	5	
subitem not at all important	○	○	○	○	○	essential

Does your paper address subitem 4b-ii?

Copy and paste relevant sections from the manuscript (include quotes in quotation marks "like this" to indicate direct quotes from your manuscript), or elaborate on this item by providing additional information not in the ms, or briefly explain why the item is not applicable/relevant for your study

Tu respuesta

5) The interventions for each group with sufficient details to allow replication, including how and when they were actually administered

5-i) Mention names, credential, affiliations of the developers, sponsors, and owners

Mention names, credential, affiliations of the developers, sponsors, and owners [6] (if authors/evaluators are owners or developer of the software, this needs to be declared in a “Conflict of interest” section or mentioned elsewhere in the manuscript).

	1	2	3	4	5	
subitem not at all important	○	○	○	○	○	essential

Does your paper address subitem 5-i?

Copy and paste relevant sections from the manuscript (include quotes in quotation marks "like this" to indicate direct quotes from your manuscript), or elaborate on this item by providing additional information not in the ms, or briefly explain why the item is not applicable/relevant for your study

"The VR software ConVRself, developed by Virtual Bodyworks SL, displays 3D scenarios and virtual human representations."

5-ii) Describe the history/development process

Describe the history/development process of the application and previous formative evaluations (e.g., focus groups, usability testing), as these will have an impact on adoption/use rates and help with interpreting results.

	1	2	3	4	5	
subitem not at all important	○	○	○	○	○	essential

Does your paper address subitem 5-ii?

Copy and paste relevant sections from the manuscript (include quotes in quotation marks "like this" to indicate direct quotes from your manuscript), or elaborate on this item by providing additional information not in the ms, or briefly explain why the item is not applicable/relevant for your study

"In line with our previous usability study [27], high SEQ scores on ConVRself indicated high usability and acceptance of the platform by people living with obesity, which means that the platform was well adapted to this population."

5-iii) Revisions and updating

Revisions and updating. Clearly mention the date and/or version number of the application/intervention (and comparator, if applicable) evaluated, or describe whether the intervention underwent major changes during the evaluation process, or whether the development and/or content was “frozen” during the trial. Describe dynamic components such as news feeds or changing content which may have an impact on the replicability of the intervention (for unexpected events see item 3b).

	1	2	3	4	5	
subitem not at all important	○	○	○	○	○	essential

Does your paper address subitem 5-iii?

Copy and paste relevant sections from the manuscript (include quotes in quotation marks "like this" to indicate direct quotes from your manuscript), or elaborate on this item by providing additional information not in the ms, or briefly explain why the item is not applicable/relevant for your study

Tu respuesta

5-iv) Quality assurance methods

Provide information on quality assurance methods to ensure accuracy and quality of information provided [1], if applicable.

	1	2	3	4	5	
subitem not at all important	○	○	○	○	○	essential

Does your paper address subitem 5-iv?

Copy and paste relevant sections from the manuscript (include quotes in quotation marks "like this" to indicate direct quotes from your manuscript), or elaborate on this item by providing additional information not in the ms, or briefly explain why the item is not applicable/relevant for your study

Tu respuesta

5-v) Ensure replicability by publishing the source code, and/or providing screenshots/screen-capture video, and/or providing flowcharts of the algorithms used

Ensure replicability by publishing the source code, and/or providing screenshots/screen-capture video, and/or providing flowcharts of the algorithms used. Replicability (i.e., other researchers should in principle be able to replicate the study) is a hallmark of scientific reporting.

	1	2	3	4	5	
subitem not at all important	○	○	○	○	○	essential

Does your paper address subitem 5-v?

Copy and paste relevant sections from the manuscript (include quotes in quotation marks "like this" to indicate direct quotes from your manuscript), or elaborate on this item by providing additional information not in the ms, or briefly explain why the item is not applicable/relevant for your study

Tu respuesta

5-vi) Digital preservation

Digital preservation: Provide the URL of the application, but as the intervention is likely to change or disappear over the course of the years; also make sure the intervention is archived (Internet Archive, webcitation.org, and/or publishing the source code or screenshots/videos alongside the article). As pages behind login screens cannot be archived, consider creating demo pages which are accessible without login.

	1	2	3	4	5	
subitem not at all important	○	○	○	○	○	essential

Does your paper address subitem 5-vi?

Copy and paste relevant sections from the manuscript (include quotes in quotation marks "like this" to indicate direct quotes from your manuscript), or elaborate on this item by providing additional information not in the ms, or briefly explain why the item is not applicable/relevant for your study

No open access URL available.

5-vii) Access

Access: Describe how participants accessed the application, in what setting/context, if they had to pay (or were paid) or not, whether they had to be a member of specific group. If known, describe how participants obtained “access to the platform and Internet” [1]. To ensure access for editors/reviewers/readers, consider to provide a “backdoor” login account or demo mode for reviewers/readers to explore the application (also important for archiving purposes, see vi).

	1	2	3	4	5	
subitem not at all important	○	○	○	○	○	essential

Does your paper address subitem 5-vii? *

Copy and paste relevant sections from the manuscript (include quotes in quotation marks "like this" to indicate direct quotes from your manuscript), or elaborate on this item by providing additional information not in the ms, or briefly explain why the item is not applicable/relevant for your study

"Two weeks later, EG1 and EG2 participants engaged in 4 VR scenarios at the hospital facilities, assisted by a team clinical researcher. Scenarios were distributed in weekly sessions, each lasting 30 minutes, over a period of 4 weeks."

"The VR system used in the study consisted of both hardware and software components. The VR hardware used was the Meta Quest 2, which is a stand-alone headset developed by Reality Labs (Meta Platforms, Inc). The main part of the hardware consists of a head-mounted display worn by the participants."

5-viii) Mode of delivery, features/functionalities/components of the intervention and comparator, and the theoretical framework

Describe mode of delivery, features/functionalities/components of the intervention and comparator, and the theoretical framework [6] used to design them (instructional strategy [1], behaviour change techniques, persuasive features, etc., see e.g., [7, 8] for terminology). This includes an in-depth description of the content (including where it is coming from and who developed it) [1],” whether [and how] it is tailored to individual circumstances and allows users to track their progress and receive feedback” [6]. This also includes a description of communication delivery channels and – if computer-mediated communication is a component – whether communication was synchronous or asynchronous [6]. It also includes information on presentation strategies [1], including page design principles, average amount of text on pages, presence of hyperlinks to other resources, etc. [1].

	1	2	3	4	5	
subitem not at all important	○	○	○	○	○	essential

Does your paper address subitem 5-viii? *

Copy and paste relevant sections from the manuscript (include quotes in quotation marks "like this" to indicate direct quotes from your manuscript), or elaborate on this item by providing additional information not in the ms, or briefly explain why the item is not applicable/relevant for your study

"The VR system used in the study consisted of both hardware and software components. The VR hardware used was the Meta Quest 2, which is a stand-alone headset developed by Reality Labs (Meta Platforms, Inc). The main part of the hardware consists of a head-mounted display worn by the participants. This head-mounted display has a vision- and inertia-based inside-out tracking system that allows precise tracking of the user’s head movements. In addition, the Meta Quest 2 comes equipped with hand controllers that enable interaction within the virtual environment. Finally, the Meta Quest 2 also incorporates a built-in processor that generates stereoscopic images and spatialized audio. The headset runs the ConVRself application, which was developed using the Unity 3D development environment (Unity Technologies)."

5-ix) Describe use parameters

Describe use parameters (e.g., intended “doses” and optimal timing for use). Clarify what instructions or recommendations were given to the user, e.g., regarding timing, frequency, heaviness of use, if any, or was the intervention used ad libitum.

	1	2	3	4	5	
subitem not at all important	○	○	○	○	○	essential

Does your paper address subitem 5-ix?

Copy and paste relevant sections from the manuscript (include quotes in quotation marks "like this" to indicate direct quotes from your manuscript), or elaborate on this item by providing additional information not in the ms, or briefly explain why the item is not applicable/relevant for your study

"Scenarios were distributed in weekly sessions, each lasting 30 minutes, over a period of 4 weeks."

5-x) Clarify the level of human involvement

Clarify the level of human involvement (care providers or health professionals, also technical assistance) in the e-intervention or as co-intervention (detail number and expertise of professionals involved, if any, as well as “type of assistance offered, the timing and frequency of the support, how it is initiated, and the medium by which the assistance is delivered”. It may be necessary to distinguish between the level of human involvement required for the trial, and the level of human involvement required for a routine application outside of a RCT setting (discuss under item 21 – generalizability).

	1	2	3	4	5	
subitem not at all important	○	○	○	○	○	essential

Does your paper address subitem 5-x?

Copy and paste relevant sections from the manuscript (include quotes in quotation marks "like this" to indicate direct quotes from your manuscript), or elaborate on this item by providing additional information not in the ms, or briefly explain why the item is not applicable/relevant for your study

"Two weeks later, EG1 and EG2 participants engaged in 4 VR scenarios at the hospital facilities, assisted by a team clinical researcher."

5-xi) Report any prompts/reminders used

Report any prompts/reminders used: Clarify if there were prompts (letters, emails, phone calls, SMS) to use the application, what triggered them, frequency etc. It may be necessary to distinguish between the level of prompts/reminders required for the trial, and the level of prompts/reminders for a routine application outside of a RCT setting (discuss under item 21 – generalizability).

	1	2	3	4	5	
subitem not at all important	○	○	○	○	○	essential

Does your paper address subitem 5-xi? *

Copy and paste relevant sections from the manuscript (include quotes in quotation marks "like this" to indicate direct quotes from your manuscript), or elaborate on this item by providing additional information not in the ms, or briefly explain why the item is not applicable/relevant for your study

"Participant data were managed and automatically distributed using Research Electronic Data Capture (REDCap; Vanderbilt University) [28,29], hosted at the Vall d’Hebron Research Institute in Barcelona, Spain"

"Furthermore, REDCap implemented an automatic alert system to remind participants to complete the questionnaires if they had not done so at the scheduled time."

5-xii) Describe any co-interventions (incl. training/support)

Describe any co-interventions (incl. training/support): Clearly state any interventions that are provided in addition to the targeted eHealth intervention, as ehealth intervention may not be designed as stand-alone intervention. This includes training sessions and support [1]. It may be necessary to distinguish between the level of training required for the trial, and the level of training for a routine application outside of a RCT setting (discuss under item 21 – generalizability.

	1	2	3	4	5	
subitem not at all important	○	○	○	○	○	essential

Does your paper address subitem 5-xii? *

Copy and paste relevant sections from the manuscript (include quotes in quotation marks "like this" to indicate direct quotes from your manuscript), or elaborate on this item by providing additional information not in the ms, or briefly explain why the item is not applicable/relevant for your study

"After completing the baseline assessment, EG1 participants underwent a 1-day MI training at the hospital facilities. The training lasted 4 hours and focused on developing basic MI skills. In addition, participants had an individual coaching session via telephone 1 week after the training. The initial in-person training session was led by an expert in MI (author PL), while the follow-up sessions were carried out by the other members of the research team."

6a) Completely defined pre-specified primary and secondary outcome measures, including how and when they were assessed

Does your paper address CONSORT subitem 6a? *

Copy and paste relevant sections from the manuscript (include quotes in quotation marks "like this" to indicate direct quotes from your manuscript), or elaborate on this item by providing additional information not in the ms, or briefly explain why the item is not applicable/relevant for your study

The primary outcomes (motivation to lose weight and exercise more) were assessed using 2 measurement tools: the RR [8] and the Stages (S-Weight) and Processes (P-Weight) of Change Questionnaires for Weight Management [30].

Eating habits:
"The Three-Factor Eating Questionnaire–Revised 18 items (TFEQ-R18) [31] is a self-report questionnaire designed to measure three aspects of eating behavior: (1) cognitive restraint (CR; 6 items), (2) uncontrolled eating (UE; 9 items), and (3) emotional eating (EE; 3 items)."
"The Eating Habits Questionnaire [33] is a self-report questionnaire with 37 items, each rated using a 5-point Likert scale ranging from never to always."

Psychological variables:

"Psychological functioning was estimated with the Hospital Anxiety and Depression Scale (HADS) [34]. The HADS is a self-report 14-item questionnaire (7 items for anxiety and 7 items for depression)."
"Body satisfaction was measured using the 10-item validated Spanish version of the Body Shape Questionnaire (BSQ-10)"
"The Modified Weight Bias Internalization Scale (WBIS-M) [37] was used to assess weight bias interiorization."
"The Cognitive Reserve Questionnaire [38] was used to measure participants’ cognitive reserve."
To measure satisfaction, acceptance, and security regarding the use of the ConVRself platform, we used the SEQ [39].

6a-i) Online questionnaires: describe if they were validated for online use and apply CHERRIES items to describe how the questionnaires were designed/deployed

If outcomes were obtained through online questionnaires, describe if they were validated for online use and apply CHERRIES items to describe how the questionnaires were designed/deployed [9].

	1	2	3	4	5	
subitem not at all important	○	○	○	○	○	essential

Does your paper address subitem 6a-i?

Copy and paste relevant sections from manuscript text

Tu respuesta

6a-ii) Describe whether and how “use” (including intensity of use/dosage) was defined/measured/monitored

Describe whether and how “use” (including intensity of use/dosage) was defined/measured/monitored (logins, logfile analysis, etc.). Use/adoption metrics are important process outcomes that should be reported in any ehealth trial.

	1	2	3	4	5	
subitem not at all important	○	○	○	○	○	essential

Does your paper address subitem 6a-ii?

Copy and paste relevant sections from manuscript text

There were no different use intensity.

6a-iii) Describe whether, how, and when qualitative feedback from participants was obtained

Describe whether, how, and when qualitative feedback from participants was obtained (e.g., through emails, feedback forms, interviews, focus groups).

	1	2	3	4	5	
subitem not at all important	○	○	○	○	○	essential

Does your paper address subitem 6a-iii?

Copy and paste relevant sections from manuscript text

Along with the questionnaires, a brief interview was conducted after each exposure to assess participants’ satisfaction with the VR experience and acceptability of ConVRself.

6b) Any changes to trial outcomes after the trial commenced, with reasons

Does your paper address CONSORT subitem 6b? *

Copy and paste relevant sections from the manuscript (include quotes in quotation marks "like this" to indicate direct quotes from your manuscript), or elaborate on this item by providing additional information not in the ms, or briefly explain why the item is not applicable/relevant for your study

No changes to trial outcomes after the trial commenced.

7a) How sample size was determined

NPT: When applicable, details of whether and how the clustering by care provides or centers was addressed

7a-i) Describe whether and how expected attrition was taken into account when

calculating the sample size

Describe whether and how expected attrition was taken into account when calculating the sample size.

1 2 3 4 5

subitem not at all important ○ ○ ○ ○ ○ essential

Does your paper address subitem 7a-i?

Copy and paste relevant sections from manuscript title (include quotes in quotation marks "like this" to indicate direct quotes from your manuscript), or elaborate on this item by providing additional information not in the ms, or briefly explain why the item is not applicable/relevant for your study

"Details regarding the sample size calculation are available in the protocol (32).". The protocol is published at BMJ Open, and referenced in the manuscript.

7b) When applicable, explanation of any interim analyses and stopping guidelines

Does your paper address CONSORT subitem 7b? *

Copy and paste relevant sections from the manuscript (include quotes in quotation marks "like this" to indicate direct quotes from your manuscript), or elaborate on this item by providing additional information not in the ms, or briefly explain why the item is not applicable/relevant for your study

A preliminary analysis was performed in the context of a conference, but not for the final publication.

8a) Method used to generate the random allocation sequence

NPT: When applicable, how care providers were allocated to each trial group

Does your paper address CONSORT subitem 8a? *

Copy and paste relevant sections from the manuscript (include quotes in quotation marks "like this" to indicate direct quotes from your manuscript), or elaborate on this item by providing additional information not in the ms, or briefly explain why the item is not applicable/relevant for your study

"Once the necessary data were collected, participants were randomly distributed in 1 of the 3 groups (EG1, EG2, and CG) using REDCap and were then informed about the outcome of the randomization. Next, they were asked to complete the online baseline assessment (T0) within the following week, which was managed by the automatic email distribution facilitated by REDCap."

8b) Type of randomisation; details of any restriction (such as blocking and block size)

Does your paper address CONSORT subitem 8b? *

Copy and paste relevant sections from the manuscript (include quotes in quotation marks "like this" to indicate direct quotes from your manuscript), or elaborate on this item by providing additional information not in the ms, or briefly explain why the item is not applicable/relevant for your study

"Once the necessary data were collected, participants were randomly distributed in 1 of the 3 groups (EG1, EG2, and CG) using REDCap and were then informed about the outcome of the randomization. Next, they were asked to complete the online baseline assessment (T0) within the following week, which was managed by the automatic email distribution facilitated by REDCap."

9) Mechanism used to implement the random allocation sequence (such as sequentially numbered containers), describing any steps taken to conceal the sequence until interventions were assigned

Does your paper address CONSORT subitem 9? *

Copy and paste relevant sections from the manuscript (include quotes in quotation marks "like this" to indicate direct quotes from your manuscript), or elaborate on this item by providing additional information not in the ms, or briefly explain why the item is not applicable/relevant for your study

"Once the necessary data were collected, participants were randomly distributed in 1 of the 3 groups (EG1, EG2, and CG) using REDCap and were then informed about the outcome of the randomization. Next, they were asked to complete the online baseline assessment (T0) within the following week, which was managed by the automatic email distribution facilitated by REDCap."

10) Who generated the random allocation sequence, who enrolled participants, and who assigned participants to interventions

Does your paper address CONSORT subitem 10? *

Copy and paste relevant sections from the manuscript (include quotes in quotation marks "like this" to indicate direct quotes from your manuscript), or elaborate on this item by providing additional information not in the ms, or briefly explain why the item is not applicable/relevant for your study

"Eligible participants were contacted via telephone by a research team member who provided information about the study. If they agreed to participate in the study, an appointment was scheduled at the hospital. During this appointment, a clinical interview was conducted by the team member to confirm the participants' eligibility. Sociodemographic and clinical variables were also collected, and weight and height measurements were taken. Once the necessary data were collected, participants were randomly distributed in 1 of the 3 groups (EG1, EG2, and CG) using REDCap and were then informed about the outcome of the randomization."

"Once the necessary data were collected, participants were randomly distributed in 1 of the 3 groups (EG1, EG2, and CG) using REDCap and were then informed about the outcome of the randomization. Next, they were asked to complete the online baseline assessment (T0) within the following week, which was managed by the automatic email distribution facilitated by REDCap."

11a) If done, who was blinded after assignment to interventions (for example, participants, care providers, those assessing outcomes) and how

NPT: Whether or not administering co-interventions were blinded to group assignment

11a-i) Specify who was blinded, and who wasn't

Specify who was blinded, and who wasn't. Usually, in web-based trials it is not possible to blind the participants [1, 3] (this should be clearly acknowledged), but it may be possible to blind outcome assessors, those doing data analysis or those administering co-interventions (if any).

1 2 3 4 5

subitem not at all important ○ ○ ○ ○ ○ essential

Does your paper address subitem 11a-i? *

Copy and paste relevant sections from the manuscript (include quotes in quotation marks "like this" to indicate direct quotes from your manuscript), or elaborate on this item by providing additional information not in the ms, or briefly explain why the item is not applicable/relevant for your study

Neither the participants nor the researchers were blinded.

11a-ii) Discuss e.g., whether participants knew which intervention was the "intervention of interest" and which one was the "comparator"

Informed consent procedures (4a-ii) can create biases and certain expectations - discuss e.g., whether participants knew which intervention was the "intervention of interest" and which one was the "comparator".

1 2 3 4 5

subitem not at all important ○ ○ ○ ○ ○ essential

Does your paper address subitem 11a-ii?

Copy and paste relevant sections from the manuscript (include quotes in quotation marks "like this" to indicate direct quotes from your manuscript), or elaborate on this item by providing additional information not in the ms, or briefly explain why the item is not applicable/relevant for your study

Tu respuesta

11b) If relevant, description of the similarity of interventions

(this item is usually not relevant for ehealth trials as it refers to similarity of a placebo or sham intervention to a active medication/intervention)

Does your paper address CONSORT subitem 11b? *

Copy and paste relevant sections from the manuscript (include quotes in quotation marks "like this" to indicate direct quotes from your manuscript), or elaborate on this item by providing additional information not in the ms, or briefly explain why the item is not applicable/relevant for your study

"In each of the 4 scenarios, participants had a self-conversation using the embodiment and body-swapping techniques. Specifically, for exposures 1, 2, and 4, participants alternated between their look-alike avatar and the avatar of a counselor. During exposure 3, they alternated between their own avatar and an avatar representing their future self—a representation depicting their future self after adopting a healthier lifestyle 5 years from the present."

"EG2 participants received a traditional counseling approach in a virtual setting. In all 4 exposures, participants were only embodied in their own look-alike avatars. First, participants engaged in a prerecorded discourse, conducted by a virtual counselor, that posed open-ended questions to which the participants responded."

12a) Statistical methods used to compare groups for primary and secondary outcomes

NPT: When applicable, details of whether and how the clustering by care providers or centers was addressed

Does your paper address CONSORT subitem 12a? *

Copy and paste relevant sections from the manuscript (include quotes in quotation marks "like this" to indicate direct quotes from your manuscript), or elaborate on this item by providing additional information not in the ms, or briefly explain why the item is not applicable/relevant for your study

"Analyses for our primary and secondary outcomes were tested with 2-level hierarchical linear models (HLMs)."

12a-i) Imputation techniques to deal with attrition / missing values

Imputation techniques to deal with attrition / missing values: Not all participants will use the intervention/comparator as intended and attrition is typically high in ehealth trials. Specify how participants who did not use the application or dropped out from the trial were treated in the statistical analysis (a complete case analysis is strongly discouraged, and simple imputation techniques such as LOCF may also be problematic [4]).

1 2 3 4 5

subitem not at all important ○ ○ ○ ○ ○ essential

Does your paper address subitem 12a-i? *

Copy and paste relevant sections from the manuscript (include quotes in quotation marks "like this" to indicate direct quotes from your manuscript), or elaborate on this item by providing additional information not in the ms, or briefly explain why the item is not applicable/relevant for your study

"Analyses were conducted using SPSS (version 29.0; IBM Corp) and RStudio (version 2022.12.0; Posit Software, PBC), with the mice package from RStudio [46] for passive multiple imputation."

12b) Methods for additional analyses, such as subgroup analyses and adjusted analyses

Does your paper address CONSORT subitem 12b? *

Copy and paste relevant sections from the manuscript (include quotes in quotation marks "like this" to indicate direct quotes from your manuscript), or elaborate on this item by providing additional information not in the ms, or briefly explain why the item is not applicable/relevant for your study

"Initial analyses involved comparisons of EG1, EG2, and CG participants on sociodemographic and clinical characteristics, adherence (dropout analysis), and assessment variables at the baseline level. Subsequent assessments examined those participants who completed the long-term follow-up assessment and those who did not on the same sociodemographic and clinical characteristics and outcome variables at baseline. Depending on variable types, various statistical tests were used: the Shapiro-Wilk test for qualitative variable distribution, a 1-way ANOVA with group as a factor for normally distributed variables, the Friedman test for nonnormally distributed variables, and the chi-square test for qualitative variables."

X26) REB/IRB Approval and Ethical Considerations [recommended as subheading under "Methods"] (not a CONSORT item)

X26-i) Comment on ethics committee approval

1 2 3 4 5

subitem not at all important ○ ○ ○ ○ ○ essential

Does your paper address subitem X26-i?

Copy and paste relevant sections from the manuscript (include quotes in quotation marks "like this" to indicate direct quotes from your manuscript), or elaborate on this item by providing additional information not in the ms, or briefly explain why the item is not applicable/relevant for your study

"This study was approved by the Clinical Research Ethics Committee and Research Projects Committee of the Vall d'Hebron University Hospital (VHUH)."

x26-ii) Outline informed consent procedures

Outline informed consent procedures e.g., if consent was obtained offline or online (how? Checkbox, etc.?), and what information was provided (see 4a-ii). See [6] for some items to be included in informed consent documents.

1 2 3 4 5

subitem not at all important ○ ○ ○ ○ ○ essential

Does your paper address subitem X26-ii?

Copy and paste relevant sections from the manuscript (include quotes in quotation marks "like this" to indicate direct quotes from your manuscript), or elaborate on this item by providing additional information not in the ms, or briefly explain why the item is not applicable/relevant for your study

"Before enrollment in the study, written informed consent was obtained from all participants. To maintain confidentiality, each participant was assigned a numerical code. No compensation was provided for participating in the study."

X26-iii) Safety and security procedures

Safety and security procedures, incl. privacy considerations, and any steps taken to reduce the likelihood or detection of harm (e.g., education and training, availability of a hotline)

1 2 3 4 5

subitem not at all important ○ ○ ○ ○ ○ essential

Does your paper address subitem X26-iii?

Copy and paste relevant sections from the manuscript (include quotes in quotation marks "like this" to indicate direct quotes from your manuscript), or elaborate on this item by providing additional information not in the ms, or briefly explain why the item is not applicable/relevant for your study

Tu respuesta

RESULTS

13a) For each group, the numbers of participants who were randomly assigned, received intended treatment, and were analysed for the primary outcome

NPT: The number of care providers or centers performing the intervention in each group and the number of patients treated by each care provider in each center

Does your paper address CONSORT subitem 13a? *

Copy and paste relevant sections from the manuscript (include quotes in quotation marks "like this" to indicate direct quotes from your manuscript), or elaborate on this item by providing additional information not in the ms, or briefly explain why the item is not applicable/relevant for your study

"In EG1, of the 24 participants, 22 (92%) received the allocated intervention, of whom 16 (73%) completed the 4 exposures. At T1, 77% (17/22) responded; and at T2 and T3, 73% (16/22) responded."

"In EG2, of the 22 participants, 21 (95%) received the allocated intervention, of whom 13 (62%) completed the 4 exposures. At T1, 67% (14/21) responded; at T2, 57% (12/21); and at T3, 62% (13/21)."

"Regarding the CG, of the 22 participants, at T1, 19 (86%) responded; at T2, 12 (54%); and at T3, 17 (77%)."

As we did an Intention-to-treat, all participants were included in the study analysis.
For precise information, see Figure 1.

13b) For each group, losses and exclusions after randomisation, together with reasons

Does your paper address CONSORT subitem 13b? (NOTE: Preferably, this is shown in a CONSORT flow diagram) *

Copy and paste relevant sections from the manuscript (include quotes in quotation marks "like this" to indicate direct quotes from your manuscript), or elaborate on this item by providing additional information not in the ms, or briefly explain why the item is not applicable/relevant for your study

"As shown in Figure 1, of the initial 94 participants assessed for the study, 19 (20%) were excluded before the randomization, resulting in 75 (80%) participants being enrolled and randomized. However, after receiving the T0 assessment, of the 75 participants, 7 (9%) decided not to continue participating; therefore, 68 (91%) participants completed the T0 assessment and were allocated to 1 of the 3 groups: EG1 (n=24, 35%), EG2 (n=22, 32%), and CG (n=22, 32%)."

"In EG1, of the 24 participants, 22 (92%) received the allocated intervention, of whom 16 (73%) completed the 4 exposures. At T1, 77% (17/22) responded; and at T2 and T3, 73% (16/22) responded."

"In EG2, of the 22 participants, 21 (95%) received the allocated intervention, of whom 13 (62%) completed the 4 exposures. At T1, 67% (14/21) responded; at T2, 57% (12/21); and at T3, 62% (13/21)."

"Regarding the CG, of the 22 participants, at T1, 19 (86%) responded; at T2, 12 (54%); and at T3, 17 (77%)."

Reasons for losses are reported in the consort figure.

13b-i) Attrition diagram

Strongly recommended: An attrition diagram (e.g., proportion of participants still logging in or using the intervention/comparator in each group plotted over time, similar to a survival curve) or other figures or tables demonstrating usage/dose/engagement.

1 2 3 4 5

subitem not at all important ○ ○ ○ ○ ○ essential

Does your paper address subitem 13b-i?

Copy and paste relevant sections from the manuscript or cite the figure number if applicable (include quotes in quotation marks "like this" to indicate direct quotes from your manuscript), or elaborate on this item by providing additional information not in the ms, or briefly explain why the item is not applicable/relevant for your study

Tu respuesta

14a) Dates defining the periods of recruitment and follow-up

Does your paper address CONSORT subitem 14a? *

Copy and paste relevant sections from the manuscript (include quotes in quotation marks "like this" to indicate direct quotes from your manuscript), or elaborate on this item by providing additional information not in the ms, or briefly explain why the item is not applicable/relevant for your study

"The timeline of the assessments is depicted in Multimedia Appendix 1. The T1 assessment took place during week 6, T2 assessment during week 7, and T3 assessment during week 10 using the same automatic email distribution as with the T0 assessment."

14a-i) Indicate if critical "secular events" fell into the study period

Indicate if critical "secular events" fell into the study period, e.g., significant changes in Internet resources available or "changes in computer hardware or Internet delivery resources"

1 2 3 4 5

subitem not at all important ○ ○ ○ ○ ○ essential

Does your paper address subitem 14a-i?

Copy and paste relevant sections from the manuscript (include quotes in quotation marks "like this" to indicate direct quotes from your manuscript), or elaborate on this item by providing additional information not in the ms, or briefly explain why the item is not applicable/relevant for your study

Tu respuesta

14b) Why the trial ended or was stopped (early)

Does your paper address CONSORT subitem 14b? *

Copy and paste relevant sections from the manuscript (include quotes in quotation marks "like this" to indicate direct quotes from your manuscript), or elaborate on this item by providing additional information not in the ms, or briefly explain why the item is not applicable/relevant for your study

Our trial was not stopped any early.

15) A table showing baseline demographic and clinical characteristics for each group

NPT: When applicable, a description of care providers (case volume, qualification, expertise, etc.) and centers (volume) in each group

Does your paper address CONSORT subitem 15? *

Copy and paste relevant sections from the manuscript (include quotes in quotation marks "like this" to indicate direct quotes from your manuscript), or elaborate on this item by providing additional information not in the ms, or briefly explain why the item is not applicable/relevant for your study

Yes. We do have a table. The table contains the demographic and clinical characteristics divided into groups at baseline.

15-i) Report demographics associated with digital divide issues

In ehealth trials it is particularly important to report demographics associated with digital divide issues, such as age, education, gender, social-economic status, computer/Internet/ehealth literacy of the participants, if known.

1 2 3 4 5

subitem not at all important ○ ○ ○ ○ ○ essential

Does your paper address subitem 15-i? *

Copy and paste relevant sections from the manuscript (include quotes in quotation marks "like this" to indicate direct quotes from your manuscript), or elaborate on this item by providing additional information not in the ms, or briefly explain why the item is not applicable/relevant for your study

In the sociodemographic table, also find reported information such as age, gender, education, nationality, marital status, employment, and related clinical variables (medical antecedents, physical and mental comorbidities).

16) For each group, number of participants (denominator) included in each analysis and whether the analysis was by original assigned groups

16-i) Report multiple "denominators" and provide definitions

Report multiple "denominators" and provide definitions: Report N's (and effect sizes) "across a range of study participation [and use] thresholds" [1], e.g., N exposed, N consented, N used more than x times, N used more than y weeks, N participants "used" the intervention/comparator at specific pre-defined time points of interest (in absolute and relative numbers per group). Always clearly define "use" of the intervention.

1 2 3 4 5

subitem not at all important ○ ○ ○ ○ ○ essential

Does your paper address subitem 16-i? *

Copy and paste relevant sections from the manuscript (include quotes in quotation marks "like this" to indicate direct quotes from your manuscript), or elaborate on this item by providing additional information not in the ms, or briefly explain why the item is not applicable/relevant for your study

"As shown in Figure 1, of the initial 94 participants assessed for the study, 19 (20%) were excluded before the randomization, resulting in 75 (80%) participants being enrolled and randomized. However, after receiving the T0 assessment, of the 75 participants, 7 (9%) decided not to continue participating; therefore, 68 (91%) participants completed the T0 assessment and were allocated to 1 of the 3 groups: EG1 (n=24, 35%), EG2 (n=22, 32%), and CG (n=22, 32%)."

16-ii) Primary analysis should be intent-to-treat

Primary analysis should be intent-to-treat, secondary analyses could include comparing only "users", with the appropriate caveats that this is no longer a randomized sample (see 18-i).

1 2 3 4 5

subitem not at all important ○ ○ ○ ○ ○ essential

Does your paper address subitem 16-ii?

Copy and paste relevant sections from the manuscript (include quotes in quotation marks "like this" to indicate direct quotes from your manuscript), or elaborate on this item by providing additional information not in the ms, or briefly explain why the item is not applicable/relevant for your study

"Analyses for our primary and secondary outcomes were tested with 2-level hierarchical linear models (HLMs). These models were implemented with group (EG1, EG2, and CG) and time (T0, T1, T2, and T3 for the RR; T0, T1, and T3 for other variables) as fixed factors and participants nested within time as a random factor. For model adjustment, we used restricted maximum likelihood as the estimation method and the scaled identity as the error covariance structure."

"An intent-to-treat (ITT) analysis was conducted using the available data of all participants for outcome estimation"

17a) For each primary and secondary outcome, results for each group, and the estimated effect size and its precision (such as 95% confidence interval)

Does your paper address CONSORT subitem 17a? *

Copy and paste relevant sections from the manuscript (include quotes in quotation marks "like this" to indicate direct quotes from your manuscript), or elaborate on this item by providing additional information not in the ms, or briefly explain why the item is not applicable/relevant for your study

Primary outcomes: "First, regarding confidence to lose weight, the HLM revealed a significant time×group effect (β=−.16; P=.02). Post hoc comparisons revealed that both EG1 and EG2 showed significant differences compared to the CG at T0 versus T1, T0 versus T2, and T0 versus T3. This notable increase in confidence to lose weight for both groups can be seen in Figure 2. Second, a significant group versus time effect for readiness to exercise more (β=−.17; P=.03) was found, with post hoc comparisons showing a significant increase for EG1 compared to CG at T0 versus T2. The significant interaction effect is represented graphically in Figure 2, where we can also see how the different group conditions evolve through the different time measures. Finally, participants from all groups had a significant improvement in their confidence to exercise more between T0 and T1 and between T0 and T3 (β=.55; P=.003) and in their readiness to lose weight between T0 and T1, T0 and T2, and T0 and T3 (β=.36; P=.01)."

Secondary outcomes:
"The HLMs revealed significant results for some TFEQ-R18 and HADS subscales, while no significant group versus time and time effects were found for the Eating Habits Questionnaire, BSQ-10, and WBIS-M. The significant interaction effect for the TFEQ-R18 and HADS subscales is represented graphically in Multimedia Appendix 5."

"The HLMs revealed a significant time×group effect for the UE (β=.71; P=.01) and EE subscales (β=.29; P=.03). Post hoc comparisons revealed consistently lower levels of UE for EG1 versus CG across all time measures, and for EE, a reduction for EG1 versus CG at T0 versus T3"

"A significant group versus time effect was found for the anxiety subscale (β=.65; P=.01). In particular, post hoc comparisons revealed greater reductions in anxiety levels between T0 and T1 for EG1 compared to EG2 and CG. For the depression subscale, only a significant time effect was found (β=1.07; P=.04), indicating a decrease in depression levels across all groups over time."

"For the SEQ, the experimental groups demonstrated high suitability scores with the VR platform (EG1: mean 59.82, SD 4.00; EG2: mean 58.43, SD 5.22; d=0.30, 95% CI −0.30 to 0.89).
Regarding the BOQ, both groups displayed average positive agreement scores, showing higher mean in EG1 than in EG2. Specifically, from the perspective of their look-alike avatar, for item 1 (looking down at the virtual body), EG1 had a mean of 2.09 (SD 1.17), while EG2 had a mean of 0.05 (SD 2.12; d=1.18, 95% CI 0.53-1.81); for item 2 (observing oneself in a virtual mirror), EG1 scored a mean of 2.34 (SD 0.70), and EG2 scored a mean of 1.53 (SD 1.58; d=0.66, 95% CI 0.05-1.26); for item 3 (perceiving body movements), EG1 scored a mean of 2.56 (SD 0.45), and EG2 scored a mean of 2.14 (SD 0.97; d=0.56, 95% CI −0.54 to 1.14); and for item 4 (recognizing oneself), EG1 scored a mean of 1.53 (SD 1.31), and EG2 scored a mean of 1.36 (SD 1.57; d=0.12; 95% CI −0.47 to 0.71). From the counselor's perspective, EG1 also demonstrated average agreement scores: (1) looking down at the virtual body: mean 1.72 (SD 1.25); (2) observing oneself in a virtual mirror: mean 1.99 (SD 0.81); and (3) perceiving body movements: mean 2.25 (SD 0.64)."

We reported Effect size and Standard Error for all primary and secondary outcomes. Confidence intervals are reported where the Cohen's d is present, and in the figures for any significant output.

17a-i) Presentation of process outcomes such as metrics of use and intensity of use

In addition to primary/secondary (clinical) outcomes, the presentation of process outcomes such as metrics of use and intensity of use (dose, exposure) and their operational definitions is critical. This does not only refer to metrics of attrition (13-b) (often a binary variable), but also to more continuous exposure metrics such as "average session length". These must be accompanied by a technical description how a metric like a "session" is defined (e.g., timeout after idle time) [1] (report under item 6a).

1 2 3 4 5

subitem not at all important ○ ○ ○ ○ ○ essential

Does your paper address subitem 17a-i?
Copy and paste relevant sections from the manuscript (include quotes in quotation marks "like this" to indicate direct quotes from your manuscript), or elaborate on this item by providing additional information not in the ms, or briefly explain why the item is not applicable/relevant for your study

Tu respuesta

17b) For binary outcomes, presentation of both absolute and relative effect sizes is recommended

Does your paper address CONSORT subitem 17b? *
Copy and paste relevant sections from the manuscript (include quotes in quotation marks "like this" to indicate direct quotes from your manuscript), or elaborate on this item by providing additional information not in the ms, or briefly explain why the item is not applicable/relevant for your study

No binary outcomes presented on this RCT

18) Results of any other analyses performed, including subgroup analyses and adjusted analyses, distinguishing pre-specified from exploratory

Does your paper address CONSORT subitem 18? *
Copy and paste relevant sections from the manuscript (include quotes in quotation marks "like this" to indicate direct quotes from your manuscript), or elaborate on this item by providing additional information not in the ms, or briefly explain why the item is not applicable/relevant for your study

No additional analyses were performed.

18-i) Subgroup analysis of comparing only users
A subgroup analysis of comparing only users is not uncommon in ehealth trials, but if done, it must be stressed that this is a self-selected sample and no longer an unbiased sample from a randomized trial (see 16-iii).

1 2 3 4 5
subitem not at all important ○ ○ ○ ○ ○ essential

Does your paper address subitem 18-i?
Copy and paste relevant sections from the manuscript (include quotes in quotation marks "like this" to indicate direct quotes from your manuscript), or elaborate on this item by providing additional information not in the ms, or briefly explain why the item is not applicable/relevant for your study

"When comparing participants who completed the long-term follow-up assessments and those who did not, significant results were found regarding the following variables: (1) age (t_{65}=−2.68; P=.009), (2) TFEQ-R18 CR (t_{66}=−2.74; P=.008), (3) Eating Habits Questionnaire total score (t_{64}=−3.23; P=.002), and (4) S-Weight (χ^2_3=16.3; P=.001). More precisely, participants with a higher mean age (46.4, SD 10.3) were more likely to complete the follow-up assessments than younger participants (mean age 39.7, SD 8.8). In addition, participants who completed all assessment points had significantly higher means in the TFEQ-R18 CR subscale and Eating Habits Questionnaire total score, and a higher percentage of them were at the maintenance stage (S-Weight), compared to those who did not complete all assessments."

19) All important harms or unintended effects in each group
(for specific guidance see CONSORT for harms)

Does your paper address CONSORT subitem 19? *
Copy and paste relevant sections from the manuscript (include quotes in quotation marks "like this" to indicate direct quotes from your manuscript), or elaborate on this item by providing additional information not in the ms, or briefly explain why the item is not applicable/relevant for your study

No harms or unintended effects were reported for any group.

19-i) Include privacy breaches, technical problems
Include privacy breaches, technical problems. This does not only include physical "harm" to participants, but also incidents such as perceived or real privacy breaches [1], technical problems, and other unexpected/unintended incidents. "Unintended effects" also includes unintended positive effects [2].

1 2 3 4 5
subitem not at all important ○ ○ ○ ○ ○ essential

Does your paper address subitem 19-i?
Copy and paste relevant sections from the manuscript (include quotes in quotation marks "like this" to indicate direct quotes from your manuscript), or elaborate on this item by providing additional information not in the ms, or briefly explain why the item is not applicable/relevant for your study

Tu respuesta

19-ii) Include qualitative feedback from participants or observations from staff/researchers
Include qualitative feedback from participants or observations from staff/researchers, if available, on strengths and shortcomings of the application, especially if they point to unintended/unexpected effects or uses. This includes (if available) reasons for why people did or did not use the application as intended by the developers.

1 2 3 4 5
subitem not at all important ○ ○ ○ ○ ○ essential

Does your paper address subitem 19-ii?
Copy and paste relevant sections from the manuscript (include quotes in quotation marks "like this" to indicate direct quotes from your manuscript), or elaborate on this item by providing additional information not in the ms, or briefly explain why the item is not applicable/relevant for your study

Tu respuesta

DISCUSSION

22) Interpretation consistent with results, balancing benefits and harms, and considering other relevant evidence
NPT: In addition, take into account the choice of the comparator, lack of or partial blinding, and unequal expertise of care providers or centers in each group

22-i) Restate study questions and summarize the answers suggested by the data, starting with primary outcomes and process outcomes (use)
Restate study questions and summarize the answers suggested by the data, starting with primary outcomes and process outcomes (use).

1 2 3 4 5
subitem not at all important ○ ○ ○ ○ ○ essential

Does your paper address subitem 22-i? *
Copy and paste relevant sections from the manuscript (include quotes in quotation marks "like this" to indicate direct quotes from your manuscript), or elaborate on this item by providing additional information not in the ms, or briefly explain why the item is not applicable/relevant for your study

"This study is focused on assessing the clinical efficacy of the ConVRself platform in tackling some of the root causes of obesity. The findings of this study confirmed our hypothesis, indicating that ConVRself with embodiment and body-swapping elements, along with MI training, significantly enhanced participants' confidence to lose weight and readiness to perform physical exercise. In addition, this intervention proved effective in reducing dysfunctional eating behaviors and anxiety compared to the other groups. Overall, these findings are consistent with previous studies on the beneficial effects of positive, instructional, and motivational self-talk for performance [48]."

Primary outcomes: "Regarding the group versus time effect on motivation to change, the results indicated that participants who used ConVRself reported a significant increase in their confidence to lose weight and a higher readiness to engage in exercise compared to the control group. These positive effects were maintained for a duration of between 1 and 4 weeks after the exposures."

Secondary outcomes: "Apart from the aforementioned improvement in eating control in EG1, the same group also experienced a notable reduction in anxiety from T0 to T1 compared to EG2 and CG. These findings align with the results of 2 RCTs conducted by Manzoni et al [65,66]. In these studies, it was demonstrated that a relaxation treatment augmented by VR was more effective in reducing anxiety and EE behaviors than traditional meditation interventions at 2-week and 3-month follow-ups among women living with obesity. It is worth mentioning that anxiety scores in EG1 decreased more after the intervention compared to EG2 and CG; however, this reduction was not sustained over time. We believe that this result can be attributed to the absence of ongoing psychological support after the intervention period with ConVRself. This finding emphasizes how patients can be highly sensitive to changes in the treatment process during the preoperative period and underscores the constant need for psychological support in all pre- and postoperative treatment phases [5]. In this context, similar to the successful application of VR in addressing various mental disorders, including anxiety [67], ConVRself has the potential to provide an additional benefit by enabling patients to cope with anxiety and develop strategies aligned with their values.
In line with our previous usability study [27], high SEQ scores on ConVRself indicated high usability and acceptance of the platform by people living with obesity, which means that the platform was well adapted to this population. In addition, as regards the body ownership of the avatars, the results obtained are similar to those reported in previous literature [25,27], which indicates that participants experienced a strong sense of body ownership over the virtual avatars. However, inconsistent outcomes were found with EG2 for the looking down at the virtual body item compared to the other body ownership evaluations. The likely reason for this anomaly is a technological problem. Whenever participants looked down while embodied in their look-alike avatars, they could only see their knees. This limited visibility of their legs was primarily due to their anatomy (body size and shape), which caused most of their legs to be out of view. EG2 participants, particularly, failed to infer that seeing their knees implied seeing their entire legs. Despite the avatar's anatomy being the same for all participants, we believe that EG1 participants were more focused on engaging in self-conversation, while EG2 participants placed greater emphasis on the physical appearance of their avatars."

22-ii) Highlight unanswered new questions, suggest future research
Highlight unanswered new questions, suggest future research.

1 2 3 4 5
subitem not at all important ○ ○ ○ ○ ○ essential

Does your paper address subitem 22-ii?
Copy and paste relevant sections from the manuscript (include quotes in quotation marks "like this" to indicate direct quotes from your manuscript), or elaborate on this item by providing additional information not in the ms, or briefly explain why the item is not applicable/relevant for your study

"Finally, the high expectation of improvement from BS may have influenced the positive outcomes; therefore, it would be necessary to corroborate the results in a population with morbid obesity but without BS expectations."

"Future research should be conducted with ConVRself as a treatment not only for people living with obesity but also for patients with mental disorders or addictive behaviors. The potential of enriching virtual self-conversation during moments of blockage in patients with artificial intelligence techniques presents an exciting future research line."

20) Trial limitations, addressing sources of potential bias, imprecision, and, if relevant, multiplicity of analyses

20-i) Typical limitations in ehealth trials
Typical limitations in ehealth trials: Participants in ehealth trials are rarely blinded. Ehealth trials often look at a multiplicity of outcomes, increasing risk for a Type I error. Discuss biases due to non-use of the intervention/usability issues, biases through informed consent procedures, unexpected events.

1 2 3 4 5
subitem not at all important ○ ○ ○ ○ ○ essential

Does your paper address subitem 20-i? *
Copy and paste relevant sections from the manuscript (include quotes in quotation marks "like this" to indicate direct quotes from your manuscript), or elaborate on this item by providing additional information not in the ms, or briefly explain why the item is not applicable/relevant for your study

"This study has several limitations. First, the high presence of physical comorbidities in the participants may have been influenced by the impact of delayed medical care and the exacerbation of conditions due to the COVID-19 pandemic [57]. This could potentially affect the generalizability of our results to the broader population. Second, a high dropout rate was observed, which made it challenging to achieve the expected adherence rate as stated in the study protocol [26]. This high dropout rate and the resulting smaller sample size may have impeded the detection of medium or small effects within the sample, particularly the potential differences among the groups. Third, the study design did not allow for a clear separation of the effects of ConVRself and the motivational training on the primary and secondary variables. The interpretations derived from our results could be attributed to either the effects of the motivational training or the virtual self-conversations, or, more likely, a combination of both. Fourth, the short follow-up period may limit the generalizability of the results. It becomes necessary to conduct a longer-term follow-up of the patients to observe whether the changes obtained with the ConVRself platform are sustained. Unfortunately, this was not feasible in this study due to time constraints imposed by the European SOCRATES project and the specific characteristics of our sample (patients on the BS waiting list). Furthermore, despite intensive basic training in MI, real competence in it demands constant and prolonged practice, potentially influencing the results. Finally, the high expectation of improvement from BS may have influenced the positive outcomes; therefore, it would be necessary to corroborate the results in a population with morbid obesity but without BS expectations."

21) Generalisability (external validity, applicability) of the trial findings
NPT: External validity of the trial findings according to the intervention, comparators, patients, and care providers or centers involved in the trial

21-i) Generalizability to other populations
Generalizability to other populations: In particular, discuss generalizability to a general Internet population, outside of a RCT setting, and general patient population, including applicability of the study results for other organizations

1 2 3 4 5
subitem not at all important ○ ○ ○ ○ ○ essential

Does your paper address subitem 21-i?
Copy and paste relevant sections from the manuscript (include quotes in quotation marks "like this" to indicate direct quotes from your manuscript), or elaborate on this item by providing additional information not in the ms, or briefly explain why the item is not applicable/relevant for your study

Tu respuesta

21-ii) Discuss if there were elements in the RCT that would be different in a routine application setting
Discuss if there were elements in the RCT that would be different in a routine application setting (e.g., prompts/reminders, more human involvement, training sessions or other co-interventions) and what impact the omission of these elements could have on use, adoption, or outcomes if the intervention is applied outside of a RCT setting.

1 2 3 4 5
subitem not at all important ○ ○ ○ ○ ○ essential

Does your paper address subitem 21-ii?
Copy and paste relevant sections from the manuscript (include quotes in quotation marks "like this" to indicate direct quotes from your manuscript), or elaborate on this item by providing additional information not in the ms, or briefly explain why the item is not applicable/relevant for your study

Tu respuesta

OTHER INFORMATION

23) Registration number and name of trial registry

Does your paper address CONSORT subitem 23? *
Copy and paste relevant sections from the manuscript (include quotes in quotation marks "like this" to indicate direct quotes from your manuscript), or elaborate on this item by providing additional information not in the ms, or briefly explain why the item is not applicable/relevant for your study

Clinicaltrials.gov (identifier NCT05094557)

24) Where the full trial protocol can be accessed, if available

Does your paper address CONSORT subitem 24? *
Cite a Multimedia Appendix, other reference, or copy and paste relevant sections from the manuscript (include quotes in quotation marks "like this" to indicate direct quotes from your manuscript), or elaborate on this item by providing additional information not in the ms, or briefly explain why the item is not applicable/relevant for your study

At BMJ Open: http://dx.doi.org/10.1136/bmjopen-2022-060822

25) Sources of funding and other support (such as supply of drugs), role of funders

Does your paper address CONSORT subitem 25? *
Copy and paste relevant sections from the manuscript (include quotes in quotation marks "like this" to indicate direct quotes from your manuscript), or elaborate on this item by providing additional information not in the ms, or briefly explain why the item is not applicable/relevant for your study

"This study was funded by the European Union's Horizon 2020 Research and Innovation Programme (951930). The funders had no role in the study design, data collection and analysis, decision to publish, or preparation of the manuscript. This study was also financially supported by the Serra Húnter Programme in the form of a grant awarded to DA."

X27) Conflicts of Interest (not a CONSORT item)

X27-i) State the relation of the study team towards the system being evaluated
In addition to the usual declaration of interests (financial or otherwise), also state the relation of the study team towards the system being evaluated, i.e., state if the authors/evaluators are distinct from or identical with the developers/sponsors of the intervention.

1 2 3 4 5
subitem not at all important ○ ○ ○ ○ ○ essential

Does your paper address subitem X27-i?
Copy and paste relevant sections from the manuscript (include quotes in quotation marks "like this" to indicate direct quotes from your manuscript), or elaborate on this item by providing additional information not in the ms, or briefly explain why the item is not applicable/relevant for your study

"MS and BS are founders of Virtual Bodyworks SL, a spin-off company of the Universitat de Barcelona. EAD was employed by Virtual Bodyworks SL. All other authors declare no other conflicts of interest."

About the CONSORT EHEALTH checklist

As a result of using this checklist, did you make changes in your manuscript? *

- ○ yes, major changes
- ◉ yes, minor changes
- ○ no

What were the most important changes you made as a result of using this checklist?

Tu respuesta

How much time did you spend on going through the checklist INCLUDING making changes in your manuscript *

It has taken around 3.5 hours

As a result of using this checklist, do you think your manuscript has improved? *

- ◉ yes
- ○ no
- ○ Otro:

Would you like to become involved in the CONSORT EHEALTH group?
This would involve for example becoming involved in participating in a workshop and writing an "Explanation and Elaboration" document

- ○ yes
- ○ no
- ○ Otro:

Any other comments or questions on CONSORT EHEALTH

Tu respuesta

STOP - Save this form as PDF before you click submit
To generate a record that you filled in this form, we recommend to generate a PDF of this page (on a Mac, simply select "print" and then select "print as PDF") before you submit it.

When you submit your (revised) paper to JMIR, please upload the PDF as supplementary file.

Don't worry if some text in the textboxes is cut off, as we still have the complete information in our database. Thank you!

Final step: Click submit !
Click submit so we have your answers in our database!

Enviar Borrar formulario

Nunca envíes contraseñas a través de Formularios de Google.

Este contenido no ha sido creado ni aprobado por Google. Notificar uso inadecuado - Términos del Servicio - Política de Privacidad

Google Formularios
